# A comparative ethnobotany of Khevsureti, Samtskhe-Javakheti, Tusheti, Svaneti, and Racha-Lechkhumi, Republic of Georgia (Sakartvelo), Caucasus

**DOI:** 10.1186/s13002-016-0110-2

**Published:** 2016-09-21

**Authors:** Rainer W. Bussmann, Narel Y. Paniagua Zambrana, Shalva Sikharulidze, Zaal Kikvidze, David Kikodze, David Tchelidze, Manana Khutsishvili, Ketevan Batsatsashvili, Robbie E. Hart

**Affiliations:** 1 William L. Brown Center, Missouri Botanical Garden, P.O. Box 299, St. Louis, Missouri 63166-0299 USA; 2 Herbario Nacional de Bolivia, Instituto de Ecología-UMSA, Campus Universitario, Cota Cota Calle 27, La Paz, Bolivia; 3 Institute of Botany and Bakuriani Alpine Botanical Garden, Ilia State University, Botanikuri St. 1, 0105 Tbilisi, Georgia; 4 4-D Research Institute, Ilia State University, 5, Cholokasvili Ave, 0162 Tbilisi, Georgia

**Keywords:** Republic of Georgia, Caucasus, Traditional knowledge, Knowledge loss, Conservation

## Abstract

**Background:**

The Republic of Georgia (Sakartvelo in Georgian language) is part of the Caucasus biodiversity hotspot, and human agricultural plant use dates bat at least 6000 years. However, little ethnobiological research has been published from the region since the 1940s. Given the lack of recent research in the region, the present study we report on plant uses in Skartvelo (Republic of Georgia), Caucasus. We hypothesized that, (1) given the long tradition of plant use, and the isolation under Soviet rule, plant use both based on homegardens and wild harvesting would be more pronounced in Georgia than in the wiser region, (2) the Soviet occupation would have had broad influence on plant use, and (3) there would still be incidence of knowledge loss despite wide plant use.

**Methods:**

Fieldwork was conducted in Khevsureti, Samtskhe-Javakheti, Tusheti, Svaneti, and Racha in July–August 2013, July–August 2014, and September–October 2015. Interviews using semi-structured questionnaires were conducted with 170 participants (80 women and 90 men) after obtaining their oral prior informed consent. All interviews were carried out in the participants’ homes and gardens by native speakers of Georgian and its local dialects (Svan, Tush, Khevsur, Psav), or, where participants spoke these as their native language, Armenian, Russian, or Greek.

**Results:**

In the present study we encountered 480 plant species belonging to 249 genera of 95 families being used in the research region. The highest number of species and of unique species were reported from the remote Tusheti-Khevsureti region. Informant consensus and number of use reports were highest for each region in the food and medicinal use categories. Of the 480 plants being used in the research region 282 species were exclusively wild-harvested, 103 were grown in homegardens, and 84 were both grown in gardens and sourced in the wild.

**Conclusions:**

Plant species, and uses, found in our study, both for Georgia in general, as well as for its regions, showed clear relations to the wider Caucasus - Asia Minor - Balkans cultural complex. However, plant use in Georgia was much more diverse than reported in other studies from Eurasia.

**Electronic supplementary material:**

The online version of this article (doi:10.1186/s13002-016-0110-2) contains supplementary material, which is available to authorized users.

## Background

Georgia is situated between latitudes 41° and 44° N, and longitudes 40° and 47° E, with an area of ca. 70000 km^2^. Georgia politically associates with European Union and takes part in all major programs of European development and cooperation. However, Georgia’s geographical location depends how the boundary between Southeastern Europe and West Asia is perceived. Most commonly, this boundary is defined as the Main Range of the Greater Caucasus. In this case, Georgia, however small, appears as a transcontinental country with its larger part located south to this divide (i.e., in Asia) and smaller but strategically important parts (Khevi, Piriketi Khevsureti, etc.) located north of the continent divide (i.e., in Europe). Therefore, Georgia is often described as Eurasian country located on the crossroads of Eastern Europe and West Asia. Georgia is bounded to the west by the Black Sea, to the north and northeast by Russian Federation, to the south by Turkey and Armenia, and to the southeast by Azerbaijan [1].

The Georgian part of the Caucasus started as the Alpine geosyncline in the late Oligocene Epoch, and the region thus reflects the same structural characteristics as the younger mountains of Europe. Therefore, the Greater Caucasus Mountains are mainly composed of Cretaceous and Jurassic rocks with the Paleozoic and Precambrian rocks in the higher regions. Structurally it represents a great anticline uplifted at the margin of the Alpine geosyncline about 25 million years ago and subsequently altered by fresh cycles of erosion and uplift. Hard, crystalline, metamorphosed rocks such as schist and gneisses, as well as pre-Jurassic granites are characteristic of the western part, while softer, Early and Middle Jurassic clayey schist and sandstones characterize the eastern part. The foots of the Greater Caucasus are built of younger limestone, sandstones, and marls. By contrast, the Lesser Caucasus Mountains are formed predominantly of the Paleogene rocks interspersed by the Jurassic and Cretaceous rocks. The youngest geological structures of Georgia are represented by the vast volcanic plateaus in the southern part of country [2–4].

Two main plain areas – the plains of Colchis and Kura-Aras are also linked to the Alpine geosyncline; the former is related to the formation of the Black Sea, the latter to that of the Caspian. The Colchis plains is mainly represented by deposits broken here and there at the foots of the mountains by the protrusions of slightly older sedimentary rocks. Younger rock also underlies the Kura-Aras Lowland. Overall, three tectonic units can be distinguished by the degree of dislocation of the Earth’s crust: (1) Fold system of the Greater Caucasus; (2) The Transcaucasian intermountain area; (3) The fold system of the Lesser Caucasus. Each of these tectonic units can be further subdivided into finer units [2–4].

Georgia’s terrain is extremely complex with steep climatic gradients. Four main units of terrain can be distinguished: (1) mountains of the greater Caucasus with peaks over 5000 m (Shkara, Babis Mta, Chanchakhi, etc.); (2) the inter-mountain plains between the Greater and Lesser Caucasus mountains; (3) the mountains of the Lesser Caucasus with peaks rarely exceed 3000 m (Mepistskaro, Kheva, Shavi Klde, Kanis Mta, Arsiani); (4) The Volcanic plateau of the Southern Georgia with elevations from 1300 to 2200 m. These primary units can be further subdivided into secondary ones [2–4].

Georgia’s climate is determined by its location within a warm temperate zone between the Black and Caspian Seas, and the complexity of its terrain in which mountain ranges and their orientation play an important part. The coastline of Georgia is 330 km long and the climate of the coastal zone is warm: the mean temperature is 4–7 °C in January and 22–23 °C in July. Precipitation is abundant (1500–2000 mm annually), especially in the southern part. At the same time, The Greater Caucasus mountains bars cold air from the north, while warm and moist air from the Black Sea spreads easily into the coastal lowlands from the west till the range of Likhi, which partly impedes further westward movement of the warm and moist air waves. In central Georgia, precipitation in mountains can be twice of that in the plains. Likewise, in the west the warm oceanic-subtropical climate can be found only at lower elevations (less than 650 m); in more elevated terrains and also to the north and east the climate becomes moderately warm. In the mountains weather conditions change to cool and wet quite steeply with increasing elevation and above 2100 m the environment becomes sub-alpine and alpine; permanent snow and ice are found above 3600 m [2–4].

### The regions

Overall the research regions cover about 18700 km^2^ (about 35 % of the currently accessible territory of the Republic of Georgia), of mostly mountainous terrain, reaching from broadleaved Colchic forests in the West and on Southern slopes, conifer forests in the East and on Northern slopes, to the nival zone.

Samtskhe-Javakheti is a region formed in the 1990s in southern Georgia from the historical provinces of Meskheti (Samtskhe), Javakheti and Tori, with Akhaltsikhe as its capital. The region comprises six administrative districts (Akhaltsikhe, Adigeni, Aspindza, Borjomi, Akhalkalak and Ninotsminda). Samtskhe-Javakheti is bordered by the regions of Adjara to the west, Guria and Imereti to the north, Shida Kartli and Kvemo Kartli to the north-east and to the east, and by Armenia and Turkey to the south and southwest. The territory of Samtskhe-Javakheti region is 6413 km2. Javakheti is located on volcanic plateau with average elevation of 1800 m. The highest peaks are Didi Abuli (3304 m), Samsari (3284 m), Godorebi (3188 m), and Patara Abuli (2801 m). The climate in the Samtskhe-Javakheti is continental, characterized by moderate precipitation and pronounced seasonal variations in temperature. The mean annual temperature for the area is 9.5 °C, with an average of −1.4 °C in January and 19.5 °C in July. Generally, the region experiences cold and occasionally snowy winters and long, but mild, summers [2–6].

Svaneti and Racha-Lechkumi are historical provinces of Georgia, located on the south-facing macro-slope of the western part of the Greater Caucasus. The Svaneti range divides the region into two depressions: Zemo (Upper) Svaneti and Kvemo (Lower) Svaneti, creating a watershed between the Enguri and Tskhenistskali basins. The region has an altitudinal gradient from 800 to 4500 m and covers 4990 km^2^. The mean temperature of the warmest months (July–August) in Svaneti decreases from +22 °C at relatively low altitudes to +7 °C to −1 °C above 3200 m, and the mean temperature of the coldest month (January) from +10 °C to −30 °C or −35 °C. The annual precipitation ranges from 1500 to 2000 mm. The vegetation of the region includes montane forest, subalpine, alpine, subnival and nival zones and corresponds to the West Caucasian, i.e. Colchic, type of the vegetation [2–6].

Pshav-Khevsureti and Tusheti are located in the main Caucasus range, with elevations from 1250 to 4493 m and cover about 7300 km^2^. The climate is generally cool. Average annual temperature is 5 °C (average temperature in July is about 13–15 °C). The annual precipitation ranges from approximately 450 to 900 mm and the precipitation mainly falls as snow. The region is bordered by Dagestan in the east, Chechnya-Ingushetia in the north and Eastern Kaheti in the south, with Tebulo (4492 m), Komito (4261 m), Dano (4174 m) and Diklosmta (4285 m) as the highest peaks. Tusheti harbors a wide variety of ecozones, and this very high biodiversity [2–6].

### Plant use history

The Caucasus is counted as one of the global biodiversity hotspots, and Georgia has its fair share of the tremendous diversity of the region [5–7], and botanical exploration of the Caucasus has a long history, yielding good recent treatments of the area’s vegetation, in particular with regard to Georgia [2, 3]. Recent legal efforts have attempted to safeguard this tremendous diversity [8].

**Fig. 1:**
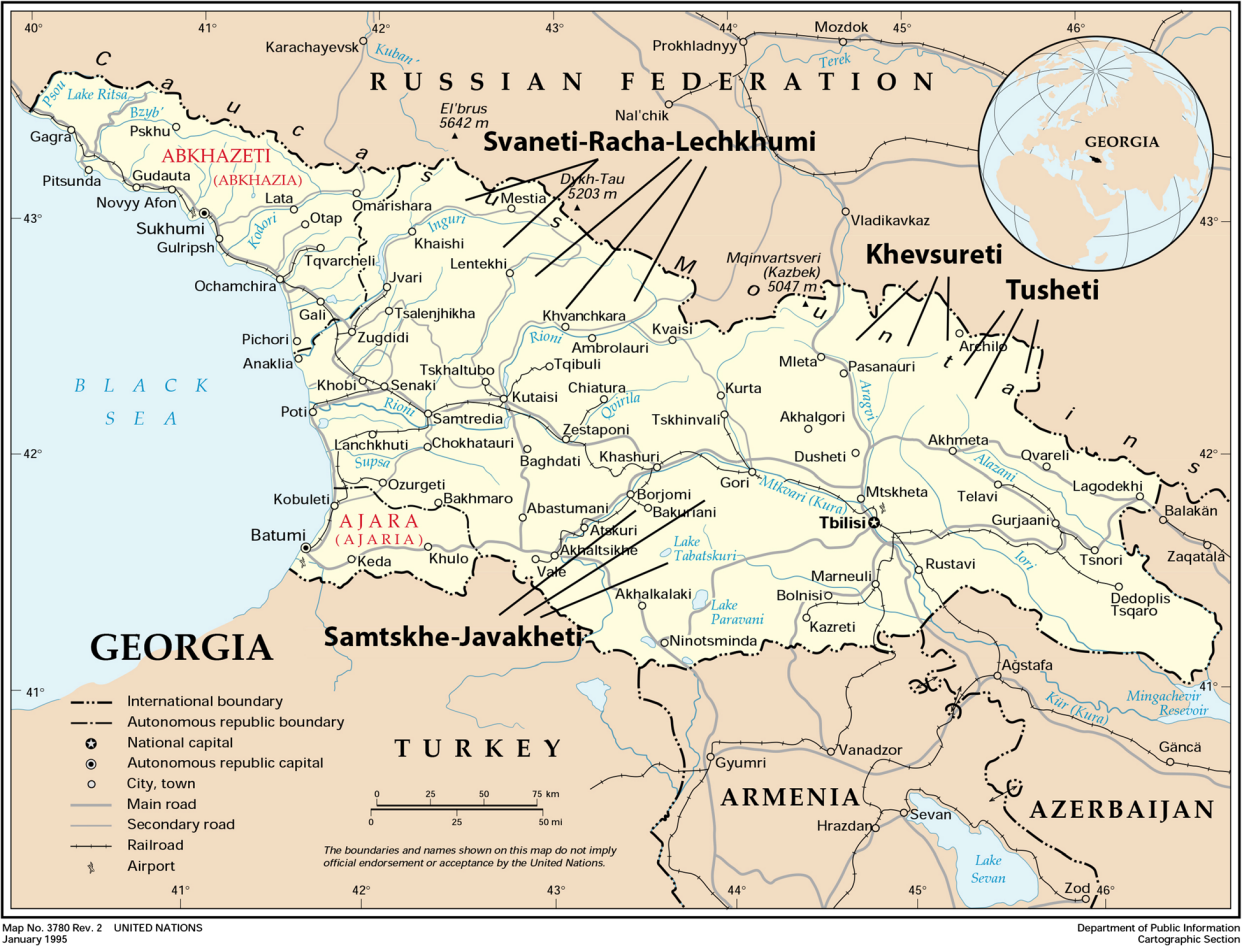
Georgia and surroundings. (based on United Nations, modified)

The territory of modern-day Georgia (Fig. 1) has been continuously inhabited since the early Stone Age, and agriculture was developed during the early Neolithic era [9]. In Georgian the name of the country is “Sakartvelo”, and “Georgia” is semantically linked to Greek (γεωργία) meaning “agriculture” [9]. Human occupation however started in the Early Pleistocene. The 1.7-Myr-old hominid fossils of Dmanisi in Southern Georgia are the earliest known hominid-site outside of Africa [10–12]. In the Late Middle Paleolithic and Early Upper Neanderthal and modern human occupation are well documented [13]. Upper Paleolithic fossils of Dzudzuana Cave include remnants of wool (*Capra caucasica*) and dyed fibers of wild flax (*Linum usitatissimum*) dated to ~36–34 Ka BP [13]. The archaeological findings from Neolithic and Early Bronze periods are rich with plant fossils and seeds of both wild species and local landraces. Seven species of cultivated wheat - *Triticum aestivum*, *T. carthlicum*, *T. compactum* Host, *T. dicoccum* Schrank, *T. macha* Dekapr. & Menabde, *T. monococcum* L., *T. spelta* L., one wild relative, *Aegilops cylindrica* Host., as well as millet - *Panicum milliaceum*, barley - *Hordeum vulgare*, Italian millet - *Setaria italica*, *Avena sativa*, *Lens ervoides* (Brignolidi & Brunhoff) Grande, and *Pisum sativum* have been discovered in Arukhlo, dating back to the 6th - 2nd millennium BC [14]. The earliest grapevine seeds indicating cultivation were excavated in southern Georgia and date to ~8.000 years BP [15]. Archaeological evidence does also exist for medicinal plant use [16], and species like *Achillea millefolium*, *Artemisia annua*, *A. absinthium*, *Centaurea jacea* and *Urtica dioica*, found in the archaeological record [16] are still found in the modern pharmacopoeia [1].

Due to its long tradition, agriculture in Georgia is characterized by a great diversity of landraces, and endemic species of crops. These show a high level of adaptation to local climatic conditions and often-high disease resistance. Early research documented this great variety [17–21], but a rapid loss of local cultivars of cereals, legumes and flax began in the 1950s with Stalinist agricultural reform [22, 23]. Despite the long cultural history, recent studies on cultivated plants are rather scarce [24, 25], and knowledge loss has been shown to extent to aggravate wolf-human conflicts [26].

*Vitis vinifera* (Vitaceae) shows its highest genetic diversity in Georgia, with about 500 cultivars known [9, 15, 27–29], and in most regions the population takes great pride to produce their own wine and share it with visitors. Hardly any house in the Georgian lowlands is without at least some grapes in its garden or backyard. Today, forty-one cultivars of grapevine are used as commercial varieties in Georgia [30], and good wine is readily available, but the history of grape cultivation and winemaking goes back millennia. Like in other parts of Europe, Georgian grapes were devastated by the *Phylloxera vastatrix* (Planchon) Signoret and after the infestation in the 1860s most Georgian grape varieties are now grafted on rootstocks of American grapes resistant to *Phylloxera*.

In the 1940s sixteen species, 144 varieties, and 150 forms of wheat (*Triticum*) were registered in Georgia [20, 21]. This diversity has however greatly diminished and most species had already disappeared by the 1960s, when introduced cultivars were favored in Soviet kolkhoz systems. At present, none of these species are sown in Georgian commercial agriculture. Pistrick et al. [24] report some traditional varieties of bread wheat in Tusheti, Meskheti, Javakheti and Svaneti. Similar diversity has been reported from nearby Turkey [31], making the region the cradle of modern European agriculture. *Hordeum vulgare* (Poaceae) is also an ancient agricultural crop in Georgia, and had particular importance in beer production, as well a function in religious rituals and traditional medicine [9, 32]. Caucasian Rye, *Secale cereale* (Poaceae) used to be cultivated in the high mountain regions of Georgia (1800–2200 m), and a large variety of landraces existed previously [33], and centered into bread and beer production, although barley was preferred for beer.

Legumes, especially peas (*Pisum sativum*), lentils (*Lens cornicularis*), chickpeas (*Cicer arietinum*), faba beans (*Vicia faba*) are still commonly grown in home gardens, and Green Pea (*Pisum sativum*) is thought to have originated in the Southern Caucasus. Traditional vegetables like garden lettuce (*Lactuca sativa*), beans (*Phaseolus vulgaris*), sweet basil (*Ocimum basilicum*), peppermint (*Mentha* x *piperita*), onions (*Allium cepa*), sugar beets (*Beta vulgaris*), spinach (*Spinaca oleracea*), carrots (*Daucus carota*), radishes (*Raphanus sativus*), turnips (*Brassica rapa* var. *rapa*), Welsh onion (*Allium fistulosum*), Amaranth (*Amaranthus viridis*), Goosefoot (*Chenopodium album*), leeks (*Allium apeloprasum*) and garlic (*Allium sativum*) are still very common throughout the region, and herbs like parsley (*Petroselinum crispum*), coriander (*Coriandrum sativum*), tarragon (*Artemisia dracunculus*), savory (*Satureja hortensis*), gardencress (*Lepidium sativum*), dill (*Anethum graveolens*), fennel (*Foeniculum vulgare*), celery (*Apium dulce*), *Allium fistulosum*, *Brassica rapa* subsp. *rapifera*, *Lathyrus sativus*, *Linum usitatissimum*, *Medicago sativa*, *Onobrychis transcaucasica*, *Pisum arvense*, *Trigonella caerulea* are cultivated almost everywhere. In addition, introduced species like zucchini (*Cucurbita pepo*), cucumber (*Cucumis sativus*), eggplant (*Solanum melongena*), marigold (*Tagetes patula*), watermelon (*Citrullus lanatus*), sunflower (*Helianthus annuus*), tomato (*Solanum lycopersicum*, pepper (*Capsicum annuum*), potato (*Solanum tuberosum*), and maize (*Zea mays*), and were found to be popular ingredients of local cuisine [1]. The maintenance of such diversity has become a priority in order to ensure global crop production [34]. *Nicotiana rustica* has been cultivated for a long time and is found in the most regions, including high mountain areas, of Georgia. *N. tabacum*, was only introduced during the Soviet period for commercial use [1].

A large number of additional species is traditionally also grown in home gardens, e.g. Sour plum (*Prunus cerasifera* var. *divaricata*) is commonly used as sauce with meat, local endemics as well as cultivars of *Pyrus* spp. are especially favored to distill liquor [35], rosehips (*Rosa canina*) are often used for tea and to make jam, and *Staphyllea pinnata* (Bladdernut) inflorescences are a favorite pickle. Many species are widely sold as medicines, giving Georgia certain potential to develop pharmaceutical industries [36].

Studies of home-gardens experienced a boom in the 1980s and 90s [37]. Home-gardens are often cited as important reservoirs for crop germplasm and as plant domestication sources [38–41]. Many studies indicate that these gardens are mostly sources of food, but that medicinal plants play only a marginal role in production [42, 43]. The cultivation of medicinal plants may help to curb the potential losses caused by destruction of natural habitats. Bussmann and Sharon [44] determined that in Peru many introduced medicinal plant species were cultivated in fields and gardens on the coast, but that the majority of native medicinal plants as collected in the wild. In contrast, especially in wider Eurasia, homegardens have been shown to be an important repository of plant diversity, and linked through complex seed exchange networks [45–49].

Given the lack of recent research in the region, n the present study we report on plant uses in Skartvelo (Republic of Georgia), Caucasus. We hypothesized that, (1) given the long tradition of plant use, and the isolation under Soviet rule, plant use both based on homegardens and wild harvesting would be more pronounced in Georgia than in the wider region, and (2) there would still be incidence of knowledge loss despite wide plant use.

## Methods

### Ethnobotanical interviews

Fieldwork was conducted in Khevsureti, Samtskhe-Javakheti, Tusheti, Svaneti, and Racha-Lechkhumi in July–August 2013, July–August 2014, and September–October 2015. Interviews using semi-structured questionnaires were conducted with 170 participants (80 women and 90 men) after obtaining their oral prior informed consent (Samtskhe-Javakheti: 34 participants (10 Armenian speakers / 23 Georgian speaker / 1 Greek/Russian speaker), Svaneti and Racha: 63 participants (all Georgian/Svan and Rachian speakers), Khevsureti, and Tusheti: 74 participants (all Georgian/Tush and Khevsuer speakers) The participants were selected by snowball sampling, trying to reach gender balance and represent members of different age (13–93 years). However, most participants were over 50 years old, as interviews targeted remote villages where only very few younger people remain. All interviews were carried out in the participants’ homes and gardens by native speakers of Georgian and its local dialects (Svan, Tush, Khevsur, Phshav), or, where participants spoke these as their native language, Armenian and in one case Greek. Russian, which all participants and interviewers were fluent in, was used as lingua franca in some interviews involving Armenian and Greek participants. Interviews were subsequently translated into English. Plants grown in the home gardens were used as prompts, while wild-collected species were free listed. In contrast to many other countries Georgia benefits from a complete flora [50–54] and a broad inventory of vernacular names in all languages spoken in Georgia, as well as the local Georgian dialects [53]. Species were identified directly in the field, using this literature, and vouchers collected and deposited in the National Herbarium of Georgia (TBI). The nomenclature of all species follows www.tropicos.org, under APGIII [55]. Collection permits were provided through the Institute of Botany, Ilia State University, Tbilisi.

### Statistical analysis

#### Distance among informants – plants and uses

Distance among informants was calculated using non-metric multi-dimensional scaling on two distance matrices: one in which columns represented plant species reported, and one in which columns represented uses reported. The resulting ordinations, in ‘plant-space’ or ‘use-space’, plot more closely together individuals who report similar plants or similar uses. We then fit different environmental vectors (community, elevation) and environmental factors (gender, region) to test how a characteristic explains the location of informants in the ordination space. To calculate a measure of significance, we compared these fits to 999 randomized shuffles of the environmental variables using the R package Vegan [56].

#### Informant consensus factor

The Informant Consensus Factor (FIC), or Informant Consensus (IC) [57] for a given Use Category was calculated as the number of use reports minus the number of taxa over the number of use reports minus one:

$$ \frac{Nur-Nt}{Nur-1} $$

#### Plant relative importance

Species were ranked by three metrics: Cultural Importance Value (CIV), the sum within species across all plant-uses of the number of informants reporting a plant-use over the number of informants reporting the plant; Use Diversity (UD), the Shannon Index of uses (calculated with the R package *vegan* [56]; and Use Value (UV), the number of reports of a species over total number of informants asked in a region [58].

#### Geographic regions and plant origin

After grouping together informants into three broad geographic regions (Svaneti-Racha, Tusheti-Khevsureti and Samtshke-Javakheti), we compared plant and use inventories across regions, and tested with the method above the fit of environmental characters onto the plant-space and use-space ordinations of informants for each region. Within these regions, the environmental factors used in our fit analysis changed slightly: we tested the fit of the environmental factor community rather than region, and for two of the regions (Samtschke-Javakheti and Tusheti-Khevsureti) our data included enough informants who had reported age that we were able to include this environmental vector. We also used this geographic grouping in computing IFC and plant relative importance metrics.

We also separately considered two groups of plant species, those grown in home-gardens and those collected from the wild. With the methods above, we analyzed regional differences among plant and use inventories as well as plant relative importance for home-garden plants, and the effect of environmental variables on individual differences, and differences in FIC for home-garden versus wild-collected plant and uses.

## Results

In the present study we encountered 480 plant species belonging to 249 genera of 95 families being used in the research region (Additional file 1). In Samtshke-Javakheti We encountered 261 useful plant species, of which 160 species were exclusively wild-collected, 81 grown in homegardens, and 20 were both grown in gardens and collected wild. In Svaneti-Racha 203 plant species were used, of which 99 species were exclusively wild-collected, 73 were grown in home-gardens, and 35 were both grown in home-gardens and collected in the wild. The highest number of useful species (317, with 197 species exclusively wild-harvested, 73 were grown in homegardens, and 47 were both grown in gardens and sourced in the wild) was found in Tusheti-Khevsureti.

**Fig. 2:**
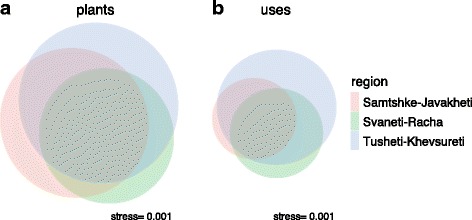
(version 2). Plants (**a**) and uses (**b**) shared among the three study regions within Georgia. Circle areas and intersections approximate the counts for each area

Plants and their uses showed mostly an overlap in the region, with a slightly wider divergence in uses. Many plant species reported were shared among all regions. However, the geographic locations of Svaneti-Racha and Tusheti-Khevsureti drove greater differences. The highest number of species and of unique species were reported from the remote Tusheti-Khevsureti region (Fig. 2a). A similar distribution was seen in plant uses, albeit with an even greater imbalance of Tusheti-Khevsureti (Fig. 2b).

**Fig. 3:**
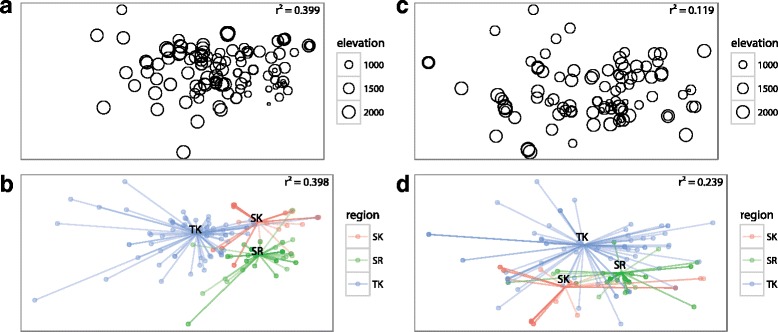
When informants from Georgia are ordered by their distance in plants reported (**a**, **b**) and in uses reported (**c**, **d**), elevation of informant community significantly fits the ordination in plantspace (B, *r* ^*2*^ = 0.399, *p* = 0.001) and in usespace (E, *r* ^*2*^ = 0.119, *p* = 0.001). Region significantly fits the ordination for both plantspace (C, *r* ^*2*^ = 0.398, *p* = 0.001) and usespace (F, *r* ^*2*^ = 0.233, *p* = 0.001)

**Fig. 4:**
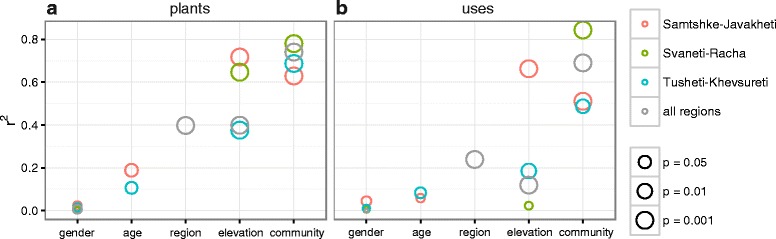
Environmental factors predicting similarity across Georgian regions are broadly similar. In four data sets (Samtshke-Kavakeli, Svaneti-Racha, Tusheti-Khevsureti, all regions), 4–5 environmental variables (informant gender, informant age (SK &TK only), region (ALL only), elevation of community, and community as categorical factor) were fit onto ordinations that positioned informants based on which plants they reported (**a**) and which uses they reported (**b**). *P*-value significance is indicated with size

Elevation of the informant communities significantly fit the ordinations in plant-space (Fig. 3a, *r*^*2*^ = 0.399, *p* = 0.001) and in use-space (Fig. 3c, *r*^*2*^ = 0.119, *p* = 0.001). Even with the significant overlaps among regions in plant and use inventories mentioned above, region also significantly fit the ordinations for both plant-space (Fig. 3b, *r*^*2*^ = 0.398, *p* = 0.001) and use-space (Fig. 3d, *r*^*2*^ = 0.233, *p* = 0.001). In contrast, informant gender was not significant in plant-space (Fig. 4a, *p* = 0.313, *r*^*2*^ = 0.007) or use-space (Fig. 4b, *p* = 0.994, *r*^*2*^ = 0).

### Informant consensus

**Fig. 5:**
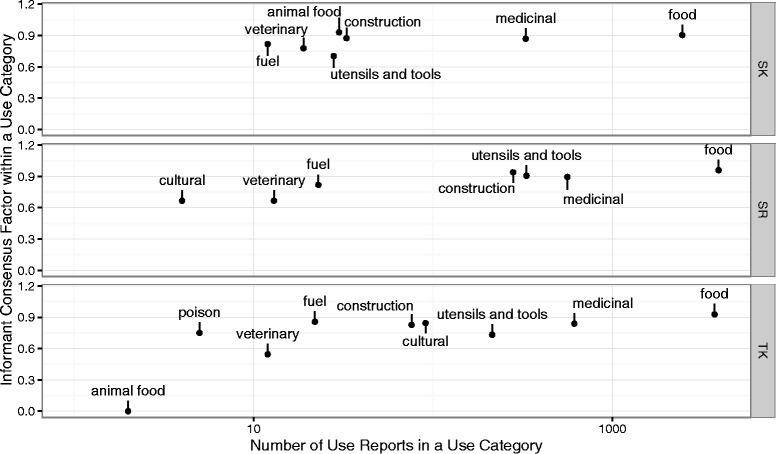
Informant consensus plotted over number of use reports for each Use Category among Georgian regions

Informant consensus and number of use reports were highest for each region in the food and medicinal use categories (Fig. 5). This consistency across regions was not as clear for other use categories: utensils and tools and construction were always high on these metrics, but were much higher in Svaneti-Racha (and, to some extent, in Tusheti-Khevsureti), perhaps reflecting the more rural nature of these areas. The cultural use category was the most various among the three regions, ranking high on both metrics in Tusheti-Khevsureti, low in Svaneti-Racha, and absent for Samstschke-Javakeli.

### Plant relative importance

**Fig. 6:**
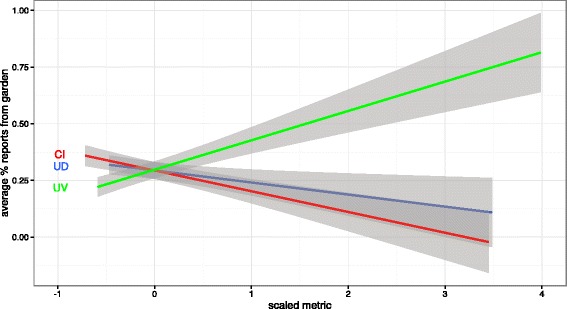
As the Use Value (UV) metric of importance (green) increases for a species, so does the likeklihood that any report of that species indicates its location as being from a Georgian home-garden. This is not the case for two other metrics of importance, Use Diversity (UD, blue) and Cultural Importance (CI, red)

The three different plant species importance ranking metrics produced quite different rankings of plant importance. Cultural Importance (CI, Table 1) prioritized species of diverse life-forms and use categories, but species with the highest CI across all of the regions were not often those with high CI within every region, or species with especially high use diversity and use value scores. Species with especially high Use Diversity (UD, Table 2) tended to be woody species. In contrast to the species with the greatest CI, species with the greatest UD did tend to also have high Use Value and CI metrics. Species with high Use Value (UV, Tables 3 and 4) tended to be common managed/domesticated species, and tended more to have high UV across regions of Georgia. In fact, with increasing UV scores, the chance that any report of a species indicated that it originated in a home-garden versus being wild-collected increased dramatically; an effect that was not seen with CI and UD (Fig. 6).

**Table 1:** The 95th percentile species ranked by Cultural Importance. Species that also appear on the 95 %ile lists for individual regions are also given. Species which are on the 95 %ile lists ranked by Use Diversity and Use Value are indicated by bold typeface in that column

Scientific name	Cultural Importance (CI)	Regional CI 95th %ile	Use Diversity	Use Value
*Juniperus hemisphaerica* C.Presl	**3.00**	SR	1.10	0.02
*Lycoperdon perlatum* Pers. / *Lycoperdon pyriforme* Schaeff.	**2.50**	SR, TK	1.05	0.06
*Betula litwinowii* Doluch.	**2.13**	SK, TK	**3.04**	0.62
*Cannabis sativa* L.	**2.00**	SR, TK	1.24	0.23
*Viola* sp.	**2.00**	SK	0.69	0.06
*Polygonum carneum* C. Koch	**2.00**	TK	1.33	0.04
*Viscum album* L.	**2.00**	TK	0.69	0.04
*Juniperus oblonga* Bieb.	**2.00**	SR	1.39	0.02
Indet sp. 28	**2.00**	SK	0.69	0.02
*Raphanus sativus* L. var. *major* (black)	**2.00**	TK	0.69	0.02
*Aethusa cynapium* L.	**2.00**	TK	0.69	0.01
*Angelica tatianae* Bordz.	**2.00**	TK	0.69	0.01
*Beta vulgaris* L. (sugar beet)	**2.00**	SR	0.69	0.01
*Lapsana grandiflora* M. Bieb	**2.00**	TK	0.69	0.01
*Sorbus terminalis* Crantz.	**2.00**	SR	0.69	0.01
*Corylus avellana* L. / *C. pontica* K. Koch.	**1.99**	SR	**1.72**	**1.15**
*Cichorium intybus* L.	**1.93**	SR	**1.63**	0.17
*Sambucus ebulus* L.	**1.93**	SR	**2.18**	0.60
*Nicotiana rustica* L.	**1.89**	TK	**1.81**	0.22
*Rosa canina* L.	**1.88**	SK	**1.69**	0.09
*Artemisia vulgaris* L.	**1.80**	SR	1.52	0.07
*Castanea sativa* Mill.	**1.79**		**1.68**	0.26
*Pinus kochiana* Klotzsch ex K. Koch	**1.78**	SK, TK	**2.39**	**1.02**
*Acer platanoides* L.	**1.75**		1.15	0.04
*Matricaria chamomilla* L.	**1.70**	SK	**1.84**	0.23

**Table 2:** The 95th percentile species ranked by Use Diversity. Species that also appear on the 95 %ile lists for individual regions are also given. Species which are on the 95%ile lists ranked by Cultural Imortance and Use Value are indicated by bold typeface in that column

Scientific name	Use Diversity (UD)	Regional UD 95th% ile	Use Value	Cultural Importance
*Betula litwinowii* Doluch.	**3.04**	SR, TK, SK	0.62	**2.13**
*Pinus kochiana* Klotzsch ex K. Koch	**2.39**	TK, SK	**1.02**	**1.78**
*Junperus sabina* L.	**2.28**	SR	0.14	1.67
*Sambucus ebulus* L.	**2.18**	SR, SK	0.6	**1.93**
*Taraxacum officinale* Wigg.	**2.14**	TK, SK	0.25	1.31
*Salix caprea* L.	**2.05**	TK	0.24	1.19
*Viburnum opulus* L.	**1.93**	TK	0.29	1.5
*Inula helenium* L.	**1.84**	TK	0.1	1.56
*Matricaria chamomilla* L.	**1.84**	SR	0.23	**1.7**
*Nicotiana rustica* L.	**1.81**	TK	0.22	**1.89**
*Viburnum lantana* L.	**1.81**	SR	0.51	1.38
*Acer trautvetteri* Medw.	**1.79**		0.11	1.29
*Nicotiana tabacum* L.	**1.79**	TK	0.14	1.64
*Carum carvi* L.	**1.78**	TK	0.34	1.2
*Corylus avellana* L. / *C. pontica* K. Koch	**1.72**	SR	**1.15**	**1.99**
*Agasyllis latifolia* (Bieb.) Boiss.	**1.71**	TK	0.5	1.37
*Bunias orientalis* L.	**1.7**	TK	0.16	1.47
*Rosa canina* L.	**1.69**	SK	0.09	**1.88**
*Castanea sativa* Mill.	**1.68**	SR	0.26	**1.79**
*Hypericum perforatum* L.	**1.68**		0.13	1.55
*Picea orientalis* (L.) Peterm.	**1.68**	SK	0.46	1.43
*Rhododendron caucasicum* Pall.	**1.66**	TK	**0.79**	1.33
*Sedum caucasicum* Boriss.	**1.64**		0.16	1.23
*Cichorium intybus* L.	**1.63**		0.17	**1.93**
*Primula macrocalyx* Bunge	**1.62**		0.12	1.29
*Vaccinium arctostaphylos* L.	**1.59**	SR	**0.77**	1.58

**Table 3:** The 95th percentile species ranked by Use Value. Species that also appear on the 95 %ile lists for individual regions are also given. Species which are on the 95 %ile lists ranked by Use Value and Cultural Importance are indicated by bold typeface in that column

Scientific name	Use Value (UV)	Regional UV 95th %ile	Use Diversity	Cultural Importance
*Malus domestica* L.	**1.55**	SR	0.32	1.12
*Pyrus communis* L.	**1.54**	SR, SK	0.24	1.05
*Coriandrum sativum* L.	**1.16**	SR, SK, TK	0.41	1.18
*Corylus avellana L*. /*C. pontica* K. Koch.	**1.15**	SR	**1.72**	**1.99**
*Allium victorialis* L.	**1.14**	SK, TK	1.05	1.56
*Rubus idaeus* L.	**1.14**	SK, TK	0.26	1.05
*Allium sativum* L.	**1.14**	SR, SK, TK	0.71	1.22
*Vitis vinifera* L.	**1.14**	SR	0.41	1.30
*Solanum tuberosum* L.	**1.11**	SK, TK	0.34	1.07
*Urtica dioica* L.	**1.03**	TK	1.54	1.34
*Pinus kochiana* Klotzsch ex K. Koch	**1.02**	SK, TK	**2.39**	**1.78**
*Beta vulgaris* L.	**0.98**	SK, TK	0.20	1.05
*Cucumis sativus* L.	**0.97**	TK	0.42	1.07
*Anethum graveolens* L.	**0.96**	TK	0.09	1.02
*Brassica oleracea* L.	**0.93**	SK, TK	0.44	1.13
*Prunus x domestica* L.	**0.93**	SR	0.46	1.19
*Daucus carota* L. ssp. *sativus*	**0.92**	TK	0.00	1.00
*Phaseolus sativus* L.	**0.90**	SK	0.00	1.00
*Prunus divaricata* Ledeb.	**0.82**	SK	0.37	1.09
*Trigonella caerulea* (L.) Ser.	**0.81**	SR	0.71	1.30
*Rosa* sp.	**0.81**	SR	1.34	1.37
*Rhododendron caucasicum* Pall.	**0.79**		**1.66**	1.33
*Vaccinium myrtillus* L.	**0.78**	TK	0.75	1.23
*Chenopodium album* L.	**0.77**		0.93	1.19
*Vaccinium arctostaphylos* L.	**0.77**	SR	**1.59**	1.58
*Triticum aestivum* L.	**0.76**		0.30	1.02

**Table 4:** Species ranked by cultural importance

Scientific name	Cultural Importance	Use Diversity	Use Value
*Solanum tuberosum* L.	1.15	0.58	1.14
*Allium victorialis* L.	1.78	1.20	1.10
*Rubus idaeus* L.	1.12	0.52	1.10
*Pinus kochiana* Klotzsch ex K. Koch	2.48	2.57	1.07
*Raphanus sativus* L. var. *major*	1.00	0.00	0.99
*Cucumis sativus* L.	1.08	0.58	0.97
*Allium sativum* L.	1.00	0.08	0.97
*Betula litwinowii* Doluch.	2.28	2.79	0.96
*Vaccinium myrtillus* L.	1.26	0.85	0.94
*Daucus carota* L. ssp. *sativus*	1.00	0.00	0.94
*Agasyllis latifolia* (Bieb.) Boiss.	1.44	1.61	0.93
*Anethum graveolens* L.	1.00	0.00	0.90
*Urtica dioica* L.	1.27	1.37	0.89
*Coriandrum sativum* L.	1.00	0.00	0.89
*Petroselinum crispum* (Mill.) Fuss.	1.00	0.24	0.87
*Brassica oleracea* L.	1.00	0.00	0.83
*Chaerophyllum caucasicum* Schischk.	1.26	1.20	0.80
*Sorbus caucasigena* Kom.	1.28	1.55	0.79
*Viburnum lantana* L.	1.44	1.23	0.79
*Beta vulgaris* L.	1.06	0.21	0.79

Considering all metrics, trees and shrubs tended to be highly important – these included wild species like *Pinus kochiana* across all metrics, *Betula litwinowii* in all regions, *Juniperus* species and *Corylus* species in Svaneti-Racha, *Sambucus ebulus*, and cultivated species like *Malus domestica* and *Pyrus communis* for UV.

### Home-garden vs. wild-collected species

Of the 480 plants being used in the research region 282 species were exclusively wild-harvested, 103 were grown in homegardens, and 84 were both grown in gardens and sourced in the wild.

**Fig. 7:**
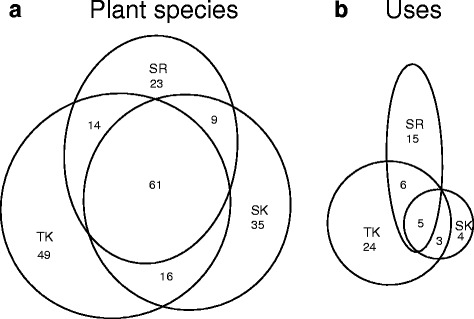
Home-garden plants (**a**) and uses (**b**) shared among the three study regions within Georgia. Ellipse areas and intersections are proportional to the count for each area

Most home-garden plants were shared among regions. Garden species showed a great deal of overlap in the region, with a slightly wider divergence in uses, in particular in Tusheti-Khevsureti. A wide number of species was shared between all regions, Svaneti and Tusheti, due to their geographic locations, showed however the highest differences, with the greatest number of species and of unique species used in the remote Tusheti-Khevsureti region (Fig. 7a). The same distribution, with an even stronger focus on Tusheti-Khevsureti occurred in plant-uses (Fig. 7b). The home-garden plants that were unique to a region were often not among the most important species; exceptions (i.e. unique and important species) include for Tusheti-Khevsureti *Raphanus sativus* var. *major* (black) and *Padus racemosa* (Table 5), and for Samtskhe-Javakheti *Mentha pulegium* (Table 6).

**Table 5:** The 95th percentile species for Tusheti-Khevsureti home gardens, ranked by Cultural Importance and by Use value. For comparison, species that also appear on the 95 %ile lists for the other two regions are shown. Species which are on the 95%ile lists are indicated by bold typeface in that column

Species	Tusheti-Khevsureti	SR	SK
Cultural Importance			
*Nicotiana rustica* L.	**2.07**	1.00	
*Raphanus sativus* L. var. *major* (black)	**2.00**		
*Cannabis sativa* L.	**2.00**	**2.00**	
*Nicotiana tabacum* L.	**1.89**	1.00	
*Juglans regia* L.	**1.57**	1.08	1.00
*Viburnum lantana* L.	**1.50**	**2.00**	
*Padus racemosa* (Lam.) Gilib.	**1.33**		
Use Value			
*Solanum tuberosum* L.	**1.18**	1.05	**1.18**
*Raphanus sativus* L. var. *major*	**1.01**	0.27	0.82
*Cucumis sativus* L.	**1.00**	0.95	1.00
*Allium sativum* L.	**1.00**	1.25	**1.15**
*Daucus carota* L. ssp. *sativus*	**0.97**	0.87	0.97
*Anethum graveolens* L.	**0.93**	1.00	1.00
*Coriandrum sativum* L.	**0.91**	**1.51**	1.06

**Table 6:** The 95th percentile species for Samtskhe-Javakheti home gardens, ranked by Cultural Importance and by Use value. For comparison, species that also appear on the 95 %ile lists for the other two regions are shown. Species which are on the 95 %ile lists are indicated by bold typeface in that column

Species	Samtshke-Javakheti	TK	SR
Cultural Importance			
*Morus alba* L.	**3.00**		1.00
*Urtica dioica* L.	**2.00**	1.00	1.00
*Mentha pulegium* L.	**1.44**		
*Sinapis arvensis* L.	**1.33**	1.00	
*Staphylea colchica* Steven	**1.33**	1.00	1.00
*Brassica oleracea* L. (Broccoli)	**1.29**		1.00
Use Value			
*Phaseolus sativus* L.	**1.53**	0.68	0.84
*Beta vulgaris* L.	**1.32**	0.81	1.00
*Prunus divaricata* Ledeb.	**1.32**	0.35	0.97
*Solanum tuberosum* L.	**1.18**	**1.18**	1.05
*Cucurbita pepo* L.	**1.18**	0.41	0.35
*Allium sativum* L.	**1.15**	**1.00**	1.25

**Fig. 8:**
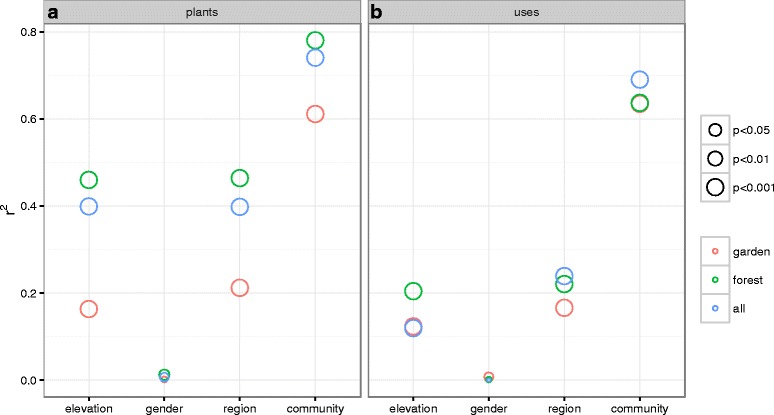
The explanatory power of environmental variables is consistenly higher for home-garden speices (green) than for wild-collected species (red) for both plants (**a**) and uses (**b**). *P*-value significance is indicated with size

Environmental factors predicting similarity across the plant species and plant uses reported by informants were broadly similar across home-garden and wild-collected species (Fig. 8): geographic and topographic factors significantly explained a portion of the variance between individuals, while gender was not significant. Home-garden plants were consistently less well explained by these environmental factors than wild-collected species (Fig. 8a).

### Informant consensus for home-garden and wild-collected species

**Fig. 9:**
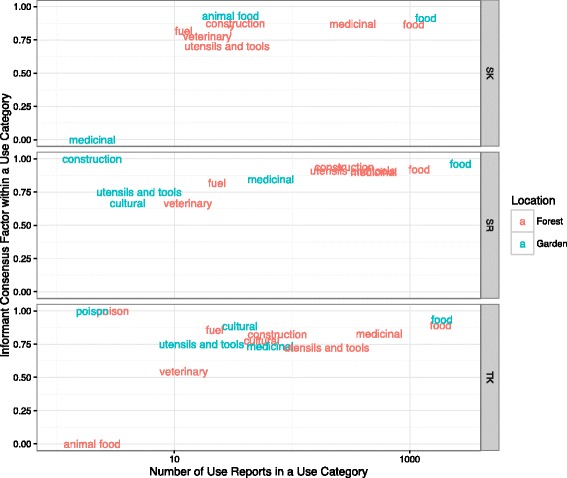
Informant consensus plotted over number of use reports for each Use Category in home garden versus forest garden plants

Although the differences between informant consensus on home garden and wild-collected plants were small, we found some interesting coincidences and differences across regions (Fig. 9). In each region, wild-collected medicinal species were consistently reported more and had higher consensus than home-garden medicinal species. In contrast, for food species, home-garden species were consistently reported more and with higher consensus than wild-collected species, although this difference was smaller than that for medicinal species (Fig. 9).

### Relative importance of home garden species

As in our consideration of plants as a whole, different metrics produced different rankings of home-garden plant importance. CI prioritized species of diverse life-forms and use categories, while species with high UV tended to be well-known food species. The influence of geography is clear especially in CI, with some plants that were unique to a region or regions, while plants with a high UV were always found across all three regions. Even so, species that fell into the 95th percentile of CI and UV for Tusheti-Khevsureti (Table 5) were not often in that tier of importance for Samtschke-Javakheli (Table 6) or Svaneti-Racha (Table 7). Home-garden plant use was consistently less well explained by these environmental factors than the use of wild-collected species (Fig. 8a). – in fact, the r^2^ value for home-garden species was about half that of wild-collected species. This may reflect a homogeneity in garden plants related to the homogeneity of the constructed niche of “domesticated” spaces vs the diversity and geographic and topographic influences on niches in more “wild” spaces.

**Table 7:** The 95th percentile species for Svaneti-Racha home gardens, ranked by Cultural Importance and by Use value. For comparison, species that also appear on the 95 % ile lists for the other two regions are shown. Species which are on the 95 %ile lists are indicated by bold typeface in that column

Species	Svaneti-Racha	TK	SK
Cultural Importance			
*Cannabis sativa* L.	**2.00**	**2.00**	
*Viburnum lantana* L.	**2.00**	**1.50**	
*Capsicum annuum* L.	**1.80**	1.00	1.00
*Foeniculum vulgare* Mill.	**1.75**	1.00	1.00
*Secale cereale* L.	**1.63**	1.00	1.00
*Allium sativum* L.	**1.53**	1.00	1.06
Use Value			
*Malus domestica* L.	**3.05**	0.54	0.59
*Pyrus communis* L.	**2.94**	0.50	1.09
*Vitis vinifera* L.	**2.41**	0.09	0.94
*Coriandrum sativum* L.	**1.51**	**0.91**	1.06
*Trigonella caerulea* (L.) Ser.	**1.44**	0.40	0.41
*Prunus avium* (L.) L.	**1.44**	0.21	0.09

## Discussion

Plant species, and uses, found in our study, both for Georgia in general, as well as for its regions, showed clear relations to the wider Mediterranean cultural complex, showing broad overlap with other studies, forming part of what Biscotti and Pieroni [59] described as “hidden Mediterranean diet”. The species number found both in all studied parts of Georgia combined, and in different sub regions, was higher than in most published studies from either the region or the wider Mediterranean and Eurasia region, where numbers of species used ranged from 44 to 330 [60–82], with the highest figure being the result of a compilation of food plants of the whole of Italy [66]. The figures from Georgia are comparable, because there, although the extension of the study area was somewhat bigger than in some of the cited comparative studies, the number of participants in each village was always very low (sometimes only 1–2), because many villages are depopulated, and the overall number of participants was either similar, or lower in Georgia. Even in close-by Dagestan, just across the Caucasus from the Georgian region of Tusheti, with a very similar cultural background, the use of wild vegetables was much lower (24 species only), although all reported uses coincided [82]. However, the lower number of participants (20, in one village only) in Dagestan might partly explain this divergence, although the field time in Dagestan was much longer, and the number of participants in Tusheti as the closest comparable region in Georgia, was only about twice as high. The much larger incidence of plant use for food in Georgia might stem from the particularly long agricultural and gardening history in the Caucasus. Interestingly, the numbers and uses of medicinal plant species, much more than food plant species, tended to coincide to other studies in the region [62, 83–91]. Again, given the comparable number of participants, these figures are comparable, although in some cases, e.g. in Turkey, only few villages in each region were surveyed. The one study that found a higher number of medicinal species in a small region in the Catalunya was published by [92]. The authors found 437 species being used.

High UV scores are found in garden rather than in wild-collected species, e.g. *Malus domestica*, *Pyrus communis*, *Coriandrum sativum*, *Corylus avellana* / *C. pontica*, *Allium victorialis*, *Vitis vinifera*; and garden species also differed much less across environmental gradients than wild-collected species. To some extent, this may reflect the wide geographic distribution of these cultivated species and their cultivated niche, in contrast to wild species with more niche divergence along geographical and topographical gradients. It also underlines the importance of Georgia as an ancient center of crop domestication and diversification and its role in the origin of many domesticated species that are globally spread today.

The use of *Rhododendron* sp. as agent to clear beer, and as medicinal tea, is rather unique, given the reports of toxicity of the species which extend from ancient Greek and Chinese sources [93, 94] to modern cases of poisoning [95]. In the main center of diversity of the genus, careful use of certain species has been reported for food and medicine [96, 97]. The protection of relatively common species like *Rhododendron caucasicum*, closely linked to its traditional use, has been shown as essential for alpine treelines often formed by *Betula litwinowii* [98].

Similaro *Rhododendron* use, a highly interesting aspect of plant use in Georgia is the use of leaves of many species generally regarded as toxic, e.g. the highly phototoxic *Heracleum* sp., toxic *Conium maculatum Galanthus* sp., *Lilium* sp. and even *Solanum tuberosum* leaves as food. This indicates a long standing experience with the local wild and cultivated flora, that allows the inhabitants of the region to make best use of all available resources, especially after long winters. Participants indicated that they were aware of the toxicity, and that such plants always needed careful preparation, e.g. long boiling with change of the water various times, and consumption only by mixing with larger quantities of other species, to avoid toxic side effects. In all cases such species were only used in early Spring, when, after a long winter, greens were very scarce. In addition, only young leaves were used. Alternatively, especially in case of *Heracleum* sp. the plant material was boiled, the water discarded, and the material then pickled with salt and vinegar, to avoid toxicity. The custom to use potentially toxic species seems a result of living conditions in isolated high altitude villages, where fresh food traditionally was scarce, and has, according to most participants, largely been abandoned, with fresh produce becoming more readily available due to better access roads, or simply by moving to the lower plains in winter.

The reported food use of use of acorns of *Quercus iberica* links Georgian customs to the wider regional food use history, as various species of *Quercus* have been reported as food from Turkey since prehistoric times [99], Very few data exist on phytochemistry and efficacy of Caucasus endemics. Research on *Vaccinium arctostaphylos* did however indicate that the species showed some efficacy as antidiabetic [100], while [101] reported on *Allium* sp. in Georgia.

## Conclusions

The process of genetic erosion of ancient crop varieties was originally of little concern for the mountain areas of Georgia, which until the 1990s acted as a repository of ancient crops. Nowadays the main reason for genetic erosion of ancient crop varieties is the demographic decline in mountain regions due to harsh economic conditions and lack of modern infrastructure [1, 23, 102–106]. The shift from ancient cultivars to modern high-yielding crops such as maize and potato, which took place in the lowland areas much earlier, began in mountain villages after the end of Soviet occupation, when local inhabitants who had been forced to the lowlands returned to their original villages. Similar changes have been reported from other former Soviet republics [106]. In addition, the rehabitation of high altitude villages has been only partial – while some families have returned at least for the summer, many villages remain in ruins. In occupied villages old household utensils like butter barrels are often to be found in storage, but not used anymore. Small bridges are still made from wood, but many other wooden household items like beautiful bed-headboards are simply discarded. Some implements, e.g. snowshoes or brooms are still maintained. Agricultural tools such as hay rakes are a common sight in abandoned barns, but more sought afar items like ox-drawn threshing sledges could only be found in museums [1]. While sheep were produced on a large scale during Soviet times, leading to widespread overgrazing, nowadays only a few scattered herds remain, and traditional wool items are getting more difficult to find, while tourist products abound along roadsides especially in the outskirts of Tbilisi and resort areas like Borjomi and Barisako. Sadly, we could only find some cultivation of *Hordeum* in Svaneti, although many participants mentioned that old landraces of wheat and barley were formerly preferred to prepare bread and beer for religious rituals. All over Georgia abandoned terraces indicate where grain was formerly grown. Many old barns still contain clay lined grain storage baskets made from *Salix* sp., which quite often contain old grains. However, essentially no grain has been grown in the surveyed high altitude regions of Georgia for decades, according to all participants recalling grain cultivation at all. One old storage chest in an abandoned barn was still half full of oats, probably harvested in the 1970s, and some wheat bran was still found in an abandoned house. Now villagers buy wheat to distil alcohol or to bake bread, or buy commercial beer making mixtures to brew their own beer [1].

The National Botanical Garden in Tbilisi runs a large seed bank and in-situ growing program for rare local species and varieties of *Triticum*, *Panicum*, and *Sorghum*, and some material is grown at the Ethnographic Museum in Tbilisi, where *Sorghum* is grown and dried and gruel with *Prunus* sauce is available to visitors, and conservation efforts to preserve endangered medicinal plant species have been started during the last decade [102].

The maintenance of home gardens in Georgia serves as socio-ecological memory, like in other regions [107, 108], and as such is an irreplaceable tool to maintain Georgian culture. In contrast to other regions, this represents not just a reflectance of growing popularity of gardening and gathering [109, 110], but cultural survival. While the great variety of plant species used in the Georgian Caucasus might provide a reservoir for food security, similar to the Balkans [110], climate change is starting to affect both natural floristic diversity and gardens both in the Caucasus as well as continent wide [111, 112].

## Additional file

Additional file 1: Plants used in Georgia. (FOREST = includes all non garden areas; GARDEN = area where species are cultivated; Arm. = Armenian; Khev. = Khevsurian; Russ. = Russian; Svan. = Svanetian; Phsh. = Pshavian). (DOCX 153 kb)
