# Supplementary material for: A comparative ethnobotany of Khevsureti, Samtskhe-Javakheti, Tusheti, Svaneti, and Racha-Lechkhumi, Republic of Georgia (Sakartvelo), Caucasus
Source: J Ethnobiol Ethnomed. 2016 Sep 21;12:43. doi: 10.1186/s13002-016-0110-2 (PMC5034577; doi:10.1186/s13002-016-0110-2)
Supplement: Additional file 1: — Plants used in Georgia. (FOREST = includes all non garden areas; GARDEN = area where species are cultivated; Arm. = Armenian; Khev. = Khevsurian; Russ. = Russian; Svan. = Svanetian; Phsh. = Pshavian). (DOCX 153 kb) [file 13002_2016_110_MOESM1_ESM.docx]

| **Family / Scientific name** | **Collection #**  **GEOETH** | **Use category (Use description)** | **Georgian Name (Transliteration)** | **Name other dialect (Transliteration other dialect)** | **Georgian variety name (Variety transliteration)** | **Part used** | **Location** |
| --- | --- | --- | --- | --- | --- | --- | --- |
| **Actinidiaceae** |  |  |  |  |  |  |  |
| *Actinidia callosa* Lindl. | 12 | Food (Human food) | კივი (Kiwi) |  |  | Fruit | Garden |
| **Adoxaceae** |  |  |  |  |  |  |  |
| *Sambucus ebulus* L. | 425 | Food (Alcohol, Human food, Khinkali, Phkhali); Medicinal (Cold, Cough, Diarrhea, Gastro intestinal system, Intestines, Liver, Lungs); Veterinary (Swollen stomach) | ანწლი (Ants’li) | გენჭვ (Gentchv Svan.), ღენღი (Genghi Svan.) |  | Flower, Fruit, Leaf, Whole plant | Forest |
| *Sambucus nigra* L. | 426 | Food (Human food) | დიდგულა (Didgula) | თოფილაი (Tophilai Svan.) |  | Fruit | Forest |
| *Viburnum lantana* L. | 488 | Cultural (Necklace, Pickled, Protection, Walking sticks); Food (Human food); Medicinal (Blood pressure, Cold, Hypertension, Inflammation, Utensils and tools, Walking sticks); Utensils and tools (Walking sticks) | უზანი (Uzani) | (T'sirchua Khev.), ალუდა (Aluda Khev.), თურსა (Tursa Tush.), ურალუზანი (Urdzani Khev.), წონწოფ (Tzontzoph Svan.) |  | Fruit, Stem | Forest, Garden |
| *Viburnum opulus L.* | 489 | Cultural (Protection); Food (Alcohol, Human food, Tea); Medicinal (Blood pressure, Cough, Heart, Lungs); Utensils and tools (Walking sticks) | უზანი (Uzani), ძახველი (Dzaxhveli) | ალუდა (Aluda Khev.), სანწეფი (Santzeph Svan.), წონწოფ (Tzontzoph Svan.) |  | Bark, Branches, Fruit, Stem | Forest |
| **Agaricaceae** |  |  |  |  |  |  |  |
| *Agaricus arvensis* Schaeff. | 14 | Food (Human food) | მინდვრის ქამა (Mindvris kama), ქამა (Kama) |  |  | Fruit | Forest, Garden |
| *Agaricus augustus* Fr. | 15 | Food (Human food) | ცხვარიო (Tskhvario) |  |  | Fruit | Forest |
| *Agaricus campestris* L. | 16 | Food (Human food) | მინდვრის სოკო (Mindvris sok'o) |  |  | Fruit | Forest |
| *Bovista* sp. | 68 | Food (Human food) | ფიჭვნარა (Pich'vnara), ფიჭვნარა სოკო (Pitshvnara soko) |  |  | Fruit | Forest |
| *Bovista* sp. | 69 | Food (Human food) | გუდაფშუტა (Gudapshut'a) |  |  | Fruit | Forest |
| *Lycoperdon* sp. | 300 | Food (Human food) | გუდაფშუტა (Gudapshut'a) |  |  | Fruit | Forest |
| *Clavatia gigantea* (Batsch) Rostk. | 114 | Food (Human food) | ფურფაშა (Purpasha), ცვარიო (Tsvario) |  |  | Fruit | Forest |
| *Coprinus comatus* (O.F.Müll.) Pers. | 119 | Food (Human food) | მერცხალა, გველის სოკო (Mertskhala, Gvelis soko) |  |  | Fruit | Forest |
| *Lycoperdon perlatum* Pers. | 298 | Food (Human food); Medicinal (Bleeding, Wounds) | მალათუ (Malatu) |  |  | Fruit, Spores | Forest |
| *Lycoperdon pyriforme* Schaeff. | 299 | Food (Human food); Medicinal (Bleeding, Wounds) | მალათუ (malatu) |  |  | Fruit, Spores | Forest |
| *Macrolepiota* sp. | 303 | Food (Human food) | წერეწო (Ts'erets'o) |  |  | Fruit | Forest |
| **Amanitaceae** |  |  |  |  |  |  |  |
| *Amanita caesarea* (Scop.) Pers. | 27 | Food (Human food) | ნიყვი (Niq'vi), წითელქუდა (Tzitelquda) |  |  | Fruit | Forest |
| *Amanita muscaria* (L.) Lam. | 28 | Food (Human food) | წითელი შხამასოკო (Tsiteli shkhamasok'o) |  |  | Fruit | Forest |
| **Amaranthaceae** |  |  |  |  |  |  |  |
| *Amaranthus palmeri* S. Watson | 29 | Food (Human food, Phkhali) | ჯიჯილაყი (jijilaqhi) |  |  | Leaf | Forest, Garden |
| *Amaranthus paniculatus* L. | 30 | Food (Human food, Phkhali) | წითელი მხალი (Ts’iteli Phkhali), წითელი ჯიჯლაყა (Ts'iteli jijlaq'a) |  |  | Leaf | Forest |
| *Amaranthus retroflexus* L. | 31 | Food (Human food, Phkhali) | ჯიჯლაყა (Jinjlaq’a) |  |  | Leaf, Stem | Forest, Garden |
| *Atriplex hortensis* L. | 53 | Food (Human food, Phkhali) | წითელი მხალი (Ts'iteli Phkhali) | თათაბო (Tatabo Tush.) |  | Leaf | Forest |
| *Beta vulgaris* L. | 56 | Food (Human food, Phkhali, Pickled) | ჭარხალი (Ch'arkhali) | (Kholhnuta Khev.), (Sokla Arm.) |  | Fruit, Leaf, Root | Garden |
| *Beta vulgaris* L. ssp. *cicla* (L.) Moq. | 57 | Food (Human food) | მანგოლდი (Mangoldi), ფოთლოვანი ჭარხალი (Photlovani charkhali), წითელი მხალი (Tsiteli phkhali) |  |  | Leaf | Garden |
| *Beta vulgaris* L. ssp. *esculenta* (Salisb.) Gürke var. *altissima* Rössig. = *Beta vulgaris saccharifera* Alef. | 58 | Food (Human food) | შაქრის ჭარხალი (Shakris ch'arkhali) |  |  | Root | Garden |
| *Beta vulgaris* L. sugar beet | 59 | Food (Human food, Pickled) |  | ხულ (Khul Svan.) |  | Leaf, Root | Forest, Garden |
| *Chenopodium album* L. | 103 | Food (Chachapuri, Human food, Phkhali, Pickled) | ნაცარქათამა (Natsarqatama) | მესგვლა (Mesgvla Svan.), მესკვა (Menshkva Svan.), ნაცარქათამა (Natzarchatari Khev.), ქათანაცარა (Qatanatsara Svan.) |  | Fruit, Leaf, Stem | Forest, Garden |
| *Chenopodium foliosum* (Moench) Asch. | 104 | Food (Human food, Phkhali, Pickled); Utensils and tools (Dye) | მათუთა (Matuta) | ძაღლთჟოლა (Dzaghltzhola Tush.) |  | leaf, Stem, Whole plant | Forest |
| *Chenopodium* sp. | 105 | Food (Pickled) |  | ნაცარქათამა (Tsatsarkatama Khev.) |  | Stem | Forest |
| **Amaryllidaceae** |  |  |  |  |  |  |  |
| *Allium apeloprasum* L. | 18 | Food (Human food) | პრასი (Prasi) | პრასა (Prasa Svan.) |  | Leaf | Garden |
| *Allium cepa* L. | 19 | Food (Human food, Pickled) | ხახვი (Khakhvi) |  |  | Bulb | Garden |
| *Allium fistulosum* L. | 20 | Food (Human food) | ჭლაკვი (Ch'lakvi) | პრასა-ხახვი (P'rasa-khakhvi), ჭაგვ (Ch'hagv Svan.) |  | Bulb, Leaf | Garden |
| *Allium kunthianum* Vved. | 21 | Food (Human food) | კლდის ხახვი (K'ldis khakhvi) | კლდისნიორა (K'ldisniora) |  | Leaf | Forest |
| *Allium porrum* L. | 22 | Food (Human food) | პრასი (P'rasi) | იმერული პრასი (Imeruli p'rasi) |  | Bulb, Stem | Garden |
| *Allium sativum* L. | 23 | Food (Human food, Pickled, Svan salt); Medicinal (Cold, Flu) | ნიორი (Niori), ნიორი (Niori) |  |  | Bulb, Flower, Leaf | Forest, Garden |
| *Allium ursinum* L. | 24 | Food (Phkhali, Pickled) | მთის ღანძილი (Mtis ghandzili), ღანძილი (Ghanzili) | ნიხანძილ (Nikhandzil Svan.) |  | Leaf | Forest |
| *Allium victorialis* L. | 25 | Food (Chachapuri, Human food, Khinkali, Phkhali, Pickled) | ღანძილი (Ghanzili) | (Masundi Arm.), დეშდვ ნივრა (Dashdven nivra Svan.), მაღდენა (Maghdena Svan.), შებუ (Shebu Khev.), შიშღილ (Shishgil Svan.), შიშღილ (Shisqil Svan.) |  | Bulb, Fruit, Leaf, Stem | Forest, Garden |
| **Anacaediaceae** |  |  |  |  |  |  |  |
| *Pistacia mutica* Fisch. & C.A. Mey. | 347 | Utensils and tools (Dye) | კევის ხე, საღსაღაჯი (Kevis khe, Saghsaghaji) |  |  | Leaf | Forest |
| **Apiaceae** |  |  |  |  |  |  |  |
| *Aethusa cynapium* L. | 13 | Food (Human food); Medicinal (Gums) | მარიამძმარა (Mariamdzmara) |  |  | Leaf | Forest |
| *Agasyllis latifolia (*Bieb.) Boiss. | 17 | Food (Chachapuri, Human food, Phkhali, Pickled); Medicinal (Anthelmintic, Asthma, Digestive system) | დუცი (Dutsi), დუცი (Dutsi) | (Gheh Svan.), ლაგი (Lagi Khev.) |  | Bark, Leaf, Petiole, Root, Stem | Forest |
| *Anethum graveolens* L. | 32 | Food (Human food, Svan salt) | კამა (K'ama) |  |  | Fruit, Leaf, Seed, Whole plant | Garden |
| *Angelica tatianae* Bordz. | 33 | Food (Human food, Pickled) | ანგელოზა (Angeloza) |  |  | Stem | Forest |
| *Anthriscus nemorosus (*M. Bieb.) Spreng. | 34 | Food (Human food, Pickled); Poison (Toxic) | ლიმი (Limi), მათუთი (Matuti) | ლიმი (Limi Svan.) |  | Flower, Fruit, Leaf, Whole plant | Forest, Garden |
| *Anthriscus sylvestris* L. | 35 | Food (Pickled) | ლიმი (Limi) | (Mandag Arm.) |  | Stem | Forest |
| *Apium graveolens* L. | 36 | Food (Human food, Phkhali) | ნიახური (Niakhuri), ნიახური (Niakhuri) |  |  | Leaf, Root, Stem | Garden |
| *Astrantia maxima* Pall. | 52 | Cultural (Decoration); Medicinal (Diuretic) | უკვდავა (Uk’vdava) |  |  | Leaf, Whole plant | Forest |
| *Carum carvi* L. | 93 | Food (Chave, Human food, Khinkali, Pickled, Spice, Svan salt); Medicinal (Diarrhea, Heartburn) | კვლიავი (K'vliavi), ძირა (Zira) | გიცრულ (Gitsrul Svan.), წყლის ქონდარი (Ts’q’lis kondari Tush.) |  | Seed | Forest, Garden |
| *Chaerophyllum aureum* L. | 97 | Food (Human food, Pickled); Medicinal (Nerves) |  | ყვასგ სვან (Qhvasg Svan.), ჭიმი (Ch’imi Tush.) |  | Root, Stem | Forest |
| *Chaerophyllum bulbosum* L. | 98 | Food (Pickled) |  | ჭიმი (Ch’imi Tush.) |  | Stem | Forest |
| *Chaerophyllum caucasicum* Schischk. | 99 | Food (Chachapuri, Human food, Khinkali, Pickled, Sats'ebai) | ღიმი (Ghimi) | (Pampara Arm.), (Shushan Arm.), (Tsipkhala Khev.), ჭიმი (Ch’imi Tush.) |  | Fruit, Leaf, Root, Stem | Forest, Garden |
| *Conium maculatum* L. | 117 | Food (Human food, Pickled) | მათუთი (Matuti) |  |  | Leaf, Stem | Forest |
| *Coriandrum sativum* L. | 120 | Food (Human food, Svan salt) | ქინძი (Kindzi) |  |  | Leaf, Seed, Stem | Garden |
| *Daucus carota* L. | 144 | Food (Human food) | შუშანა (Shushana) |  |  | Root | Forest |
| *Daucus carota* L. ssp. sativus | 145 | Food (Human food) | სტაფილო (St'apilo) | (Markowka Arm.) |  | Leaf, Root | Garden |
| *Eryngium caeruleum* M. Bieb. | 152 | Medicinal (Tea) | ლურჯი ნარი (Lurji nari) | (Tapli Arm.) |  | Leaf | Forest |
| *Falcaria sioides* Asch. | 155 | Food (Pickled) | კოფრჩხილა (K’oprchkhila) |  |  | Stem | Forest |
| *Foeniculum vulgare* Mill. | 159 | Food (Human food, Svan salt) | ცერეცო (Tseretso) |  |  | Bulb, Leaf, Root, Stem | Garden |
| *Heracleum alpinum* L. | 202 | Medicinal (Haemorrhoids) | დიყი (Diq'i) |  |  | Leaf, Seed, Stem | Forest |
| *Heracleum asperum* M. Bieb. | 203 | Food (Human food, Pickled, Sats'ebai); Medicinal (Toothache) | შუპყა (Shup'q'a), შუპყაი (Shupq’a) |  |  | Leaf, Root, Stem | Forest |
| *Heracleum leskovii* Grossh. | 204 | Food (Pickled) | შუპყაი (Shupq’a) |  |  | Stem | Forest |
| *Heracleum mantegazzianum* Sommier & Levier | 205 | Medicinal (Cancer) | დიყი (Diq'i) | ჩიჩვა (Chichva Svan.) |  | Root | Forest |
| *Heracleum* sect. *villosum* | 206 | Food (Pickled) | თეთრი დიყი (Tetri diq'i) | ლაგი (Lagi) |  | Stem | Forest |
| *Heracleum sosnowskyi* Manden | 207 | Food (Chave, Human food, Pickled, Sats'ebai); Medicinal (Cancer, Tumors) | დიყი (Diq'i) | ქეხი (Qekhi Arm.), ჩიჩვა (Chichva Svan.) |  | Leaf, Root, Seed, Stem | Forest, Garden |
| *Heracleum wilhelmsii* Fisch. & Ave-Lall | 208 | Food (Human food, Pickled); Medicinal (Stomach) | დიყი (Diq'i), დიყი (Marts'q'vi) | ქეხი (Quekhi Arm.) |  | Root, Stem | Forest |
| *Hippomarathrum microcarpum* Petrov. | 210 | Food (Pickled); Medicinal (Cold) | ქარქვეტა (Karkvet’a) |  |  | Fruit, Stem | Forest |
| *Hippomarathrum crispum* (Pers.) Boiss. | 219 | Food (Human food) | ქარქვეტა (Marts'q'vi) |  |  | Stem | Forest |
| *Levisticum officinale* W.D.J. Koch | 292 | Food (Human food) | ცისკარა (Tsisk'ara) |  |  | Leaf | Garden |
| *Libanotis transcaucasica* Schischk. | 293 | Food (Chave, Human food, Pickled, Sats'ebai) | სასუქა (Sasuka), სასუქა (Sasukai) |  |  | Leaf, Stem | Forest |
| *Ligusticum alatum* Spreng. | 294 | Food (Human food, Sats'ebai) | მარიამა (Mariamdzmara) |  |  | Leaf | Forest, Garden |
| *Petroselinum crispum* (Mill.) Fuss. | 337 | Food (Human food, Tea) | ოხრახუში (Okhrakhushi) |  |  | Leaf, Seed, Whole plant | Garden |
| **Araceae** |  |  |  |  |  |  |  |
| *Arum albispatum* Stev. ex Ledeb. | 46 | Food (Human food) |  | ქალაკოდა (Qalakoda Svan.) |  | Leaf | Forest |
| *Arum orientale* M. Bieb. | 47 | Food (Phkhali); Medicinal (Cancer) | დათვფეხა (Datvphekha), ქალაკოდა (Qalakoda) |  |  | Leaf | Forest |
| **Asparagaceae** |  |  |  |  |  |  |  |
| *Asparagus* sp. | 49 | Food (Human food) | სატაცური (Sat'acuri) |  |  | Stem | Forest |
| *Ruscus hypophyllum* L. | 417 | Food (Human food) | ძმერხლი (Dzmerkhli) |  |  | Fruit | Forest |
| **Aspleniaceae** |  |  |  |  |  |  |  |
| *Asplenium trichomanes* L. | 50 | Veterinary (Urine retention) | მამასწარა (Mamasts'ara) |  |  | Whole plant | Forest |
| **Asteraceae** |  |  |  |  |  |  |  |
| *Achillea grandiflora* M. Bieb. | 7 | Food (Human food) | ჯორთკუდა (Jortk’uda) |  |  | Leaf | Forest |
| *Achillea micrantha* M. Bieb. | 8 | Medicinal (Wounds) | ფარსმანდუკი (Parsmanduk’i) | მელაკუდა (Melakuda Tush.) |  | Leaf, Whole plant | Forest |
| *Achillea millefolium* L. | 9 | Food (Chachapuri, Tea); Medicinal (Anti-inflammatory, Cholagogic, Diuretic, Inflammation, Kidneys, Liver, Panacea, Sore throat, Stomach, Ulcers, Wounds); Utensils and tools (Dye) | ფარსმანდუკი (Parsmanduk’i) | (Khazarateig Arm.), Мелетник (Meletnik Arm.), მელაკუდა (Melik’uda Tush.), წყლულის ბალახი ( Ts’q’lulis balakhi Tush.) |  | Flower, Leaf, Whole plant | Forest, Garden |
| *Achillea nobilis* L. | 10 | Medicinal (Rheumatism, Wounds) | ტილჭირი (T’ilch’iri), ფარსმანდუკი (Parsmanduk’i) | მელაკუდა (Melik’uda Tush.) |  | Leaf, Root, Whole plant | Forest |
| *Achillea ptarmicifolia* (Willd.) Rupr. ex Heimerl | 11 | Medicinal (Wounds) | ველური ტარხუნა (Veluri tarkhuna) |  |  | Leaf | Forest |
| *Arctium lappa* L. | 37 | Food (Human food, Phkhali, Pickled); Medicinal (Antibiotic) | ძირხვენა (Dzirkhvena), ძირხვენა (Dzirkhvena) | (Graduk Arm.) |  | Flower, Leaf, Root, Stem | Forest |
| *Artemisia absinthium* L. | 41 | Food (Human food); Medicinal (Bedwetting in children, Cold, Flu, Sore throat); Veterinary (Fever) | აბზინდა (Abzinda), გიეში (Gieshi) | გიეში (Gieshi), გიეში (Gieshi Tush.) |  | Leaf, Whole plant | Forest, Garden |
| *Artemisia annua* L. | 42 | Medicinal (Insect repellant, Wounds); Veterinary (Wounds cattle) | უჯანგარი (Ujangari) |  |  | Leaf | Forest |
| Artemisia dracunculus L. | 43 | Food (Human food, Spice) | ტარხუნა (T’arkhuna) |  |  | Fruit, Leaf, Root | Garden |
| *Artemisia* sp. | 44 | Utensils and tools (Brooms) | ავშანი (Avshani) |  |  | Stem | Forest |
| *Artemisia vulgaris* L. | 45 | Food (Human food, Sats'ebai); Medicinal (Diuretic, Gallbladder, Lice) | ჯორთკუდა (Jortk’uda) | ველური ტარხუნა (Veluri T’arkhuna Svan.) |  | Leaf | Forest |
| *Bidens tripartida* L. | 63 | Food (Human food); Medicinal | ორკბილა (Ork'bila) | череда (Ch'ereda Russ.) |  | Fruit | Forest |
| *Calendula officinalis* L. | 81 | Medicinal (Wounds) | გულყვითელა, ნარგიზელა (Gulq'vitela, nargizela) |  |  | Flower | Garden |
| *Chrysanthemum leucanthemum* L. | 106 | Medicinal (Heart) | წყლულის წამალი (Ts’q’lulis tsamali) |  |  | Leaf | Forest |
| *Cichorium intybus* L. | 108 | Food (Sats'ebai); Medicinal (Diabetes, Gallbladder, Gastro intestinal system, Oral inflammation) | ხაპრაი (Khap’arai) | ვარდკაჭაჭა (Vardkach’ach’a Svan.) |  | Leaf, Root | Forest |
| *Cirsium* sp. | 109 | Food (Sats'ebai); Medicinal (Hemorrhoides) | ნარი (Nari) | (Shafalukh Arm) |  | Leaf | Forest |
| *Eruca sativa* Mill. | 151 | Food (Human food) | რუკულა (Ruk'ula) |  |  | Leaf | Garden |
| *Helianthus annuus* L. | 198 | Food (Human food) | მზესუმზირა (Mzesumzira) |  |  | Seed | Garden |
| *Helianthus tuberosus* L. | 199 | Food (Human food) | მიწავაშლა (Mits'avashla) |  |  | Leaf | Garden |
| *Helichrysum arenarium* (L.) Moench | 200 | Medicinal (Gallbladder, Gastro intestinal system, Heartburn, Liver, Tea) | ნეგო (Nego) | (Antaran tsagik Arm), ნეგი (Negi) |  | Flower, Leaf | Forest |
| *Inula helenium* L. | 270 | Cultural (Smoking); Medicinal (Asthma, Cough, Gastro intestinal system, Panacea, Respiratory tract); Veterinary (Pig plague) | კულმუხო (Kulmukho) |  |  | Leaf, Root | Forest |
| *Lactuca sativa* L. | 280 | Food (Human food) | მწვანე სალათა (Mtsvane salata), სალათა (Salata) | (Berdznuli salata) |  | Leaf | Garden |
| *Lactuca sativa* L. "greek" | 281 | Food (Human food) | მწვანე სალათა (Mtsvane salata) |  |  | Leaf | Garden |
| *Lactuca serriola* L. | 282 | Food (Human food) | ღორის ქადა (Ghoris qada) | ხარნუყა (Kharnuq’a Tush.) |  | Leaf | Garden |
| *Lapsana communis* L. | 284 | Food (Human food, Phkhali) | ვაზისძირა (Vazisdzira) |  |  | Leaf | Forest |
| *Lapsana grandiflora* M. Bieb | 285 | Food (Human food, Soup) | მწარე ხარნუყა (Mts'are kharnuq'a) |  |  | Leaf | Forest |
| *Matricaria chamomilla* L. | 310 | Food (Chave, Tea); Medicinal (Cough, Diuretic, Gastro intestinal system, Intestines, Stomach, Toothache, Wounds) | გვირილა (Gvirila) | Ромашка (Romashka Russ.) |  | Leaf, Whole plant | Forest |
| *Petasites vulgaris* Desf. | 336 | Food (Chave, Human food, Phkhali, Pickled) | ბუერა (Buera) | ბურღვილ (Burghvil Svan.) |  | Leaf, Stem | Forest, Garden |
| *Pyrethrum parthenifolium* Willd. | 380 | Medicinal (Flu, Inflammation, Oral inflammation, Toothache) | გვირილა (Gvirila) |  |  | Leaf | Forest |
| *Pyrethrum roseum* (Adams) M. Bieb. | 381 | Medicinal (Wounds) | სარწყილა (Sarts’q’ila) |  |  | Leaf | Forest |
| *Pyrethrum* sp. | 382 | Medicinal (Toothache) | გვირილა (Gvirila) |  |  | Leaf | Forest |
| *Serratula quinquefolia* Bieb. ex Willd. | 435 | Food (Phkhali, Pickled) | საღვერავი, ირმისმხალა (Irmismkhala) | ნადირისფხალი (Nadiris Phkhali), ნადირისფხალი (NadirisPhkhali) |  | Stem | Forest |
| *Sonchus asper* (L.) Hill. | 443 | Food (Human food, Phkhali) | ღიჭა (Ghich'a) |  |  | Leaf | Forest, Garden |
| *Tagetes patula* L. | 457 | Food (Human food, Svan salt) | ზაფრანა (Zaprana), ყვითელი ყვავილი - “იმერული ზაფრანა” (Qhviteli qhvavili "Imeruli zaphrana"), ხავერდა (Khaverda) | გულყვითელა (Gulqhvitela Svan.), იმერული ზაფრანა (Imeruli zaprana) |  | Flower, Fruit, Leaf, Root, Seed | Forest, Garden |
| *Taraxacum confusum* Schischk. | 458 | Food (Chave, Phkhali) | საღვიძლა (Saghvidzla) | ბურბუშელა (Burbushela Tush.) |  | Leaf | Forest |
| *Taraxacum officinale* Wigg. | 459 | Cultural (Whistles); Food (Chave, Human food, Phkhali, Tea); Medicinal (Diuretic, Gallstones, Laxative, Liver, Oral inflammation, Toothache) | საღვიძლა (Saghvidzla) | (Sajarai Svan.), ნაგურელა (Nagurela Svan.), ფანდურპაპაი (Pandurpapai Tush.) |  | Leaf, Root, Stem | Forest |
| *Tragopogon* sp. | 468 | Food (Human food, Pickled) | ფამფარა (Pampara) | (Sindz Arm.) |  | Leaf, Root, Stem | Forest, Garden |
| *Tussilago farfara* L. | 476 | Food (Tea); Medicinal (Arthritis, Bronchitis, Cold, Cough, Expectorant, Headache, Lungs, Vasodilation) | ვირისტერფა (Virist'erpa) |  |  | Leaf, Whole plant | Forest |
| **Bankeraceae** |  |  |  |  |  |  |  |
| *Hydnum repandum* Fr. | 216 | Food (Human food) | ირმისტუჩა (Irmist'ucha) |  |  | Fruit | Forest |
| *Sarcodon imbricatus* (L.) P. Karts. | 427 | Food (Human food) | ირემა სოკო (Irema-so'ko) |  |  | Fruit | Forest |
| **Berberidaceae** |  |  |  |  |  |  |  |
| *Berberis vulgaris* L. | 55 | Food (Human food, , PhkhaliTea, Tqhemali); Medicinal (Gallbladder, Hypertension, Liver); Utensils and tools (Dye) | კოწახური (K’otsakhuri), კოწახური (K'ots'akhuri) | გოცხილ (Gotskhil Svan.), ესკალძმარა (Esholtsmana Khev.) |  | Fruit, Leaf, Root | Forest, Garden |
| **Betulaceae** |  |  |  |  |  |  |  |
| *Alnus barbata* C.A. Mey. | 26 | Construction (Timber); Food (Tea); Fuel (Firewood); Utensils and tools (Dye, Furniture, Sleds, Tool handles) | თხმელა (Tkhmela), მურყანი (Murq’ani) | ბელყაც (Belqhats Svan.) |  | Bark, Leaf, Stem | Forest |
| *Betula litwinowii* Doluch. | 60 | Construction (Posts, Timber); Food (Human food); Fuel (Firestarter, Firewood); Medicinal (Arthritis, Cold, Dandruff, Goiter, Hair loss, Panacea, Toothache, Wounds); Utensils and tools (Beer ladle, Bowls, Brooms, candlesticks, Carts, Cups, Dippers, Furniture, Household utensils, Mortars, Plows, Sleds, Spinning wheels, Spoons, Tool handles, Trays, Vessels for alcohol, Walking sticks, Yokes) | არყი (Arq'i) | ბელყაც (Belqhats Svan.), ჟახვარ (Zhakhvar Svan.) |  | Bark, Branches, Fruit, Juice, Leaf, Root, Stem | Forest |
| *Betula pendula* Roth | 61 | Medicinal (Big Heart) | არყი (Arq'i), მეჭეჭიანი არყი (Mech'ech'iani arq'i) |  |  | Leaf | Forest |
| *Betula raddeana* Trautv | 62 | Construction (Timber); Utensils and tools (Household utensils) | არყი (Arq'i), შავი არყი (Shavi arq'i) |  |  | Stem | Forest |
| *Carpinus caucasica* Grossh. | 92 | Construction (Timber); Fuel (Firewood); Utensils and tools (Furniture, Sleds, Tool handles) | რცხილა (Rtskhila), ჯაგრცხილა (Jagrcxila) | ცხვიმრა (Tskhvimra Svan.) |  | Stem | Forest |
| *Corylus avellana* L. | 125 | Construction (Fences, Timber); Food (Human food, Phkhali); Medicinal (Cough, Gangrene); Utensils and tools (Baskets, Carts, Furniture, Household utensils, Rope, Sleds, Tool handles, Walking sticks) | თხილი (Tkhili), | შდიხ (Shdikh Svan.) | (Khaka Svan.), (Nemsa Svan.) | Branches, Fruit, Leaf, Stem | Forest, Garden |
| *Corylus pontica* K. Koch. | 126 | Construction (Fences, Timber); Food (Human food, Phkhali); Medicinal (Cough, Gangrene); Utensils and tools (Baskets, Carts, Furniture, Household utensils, Rope, Sleds, Tool handles, Walking sticks) | თხილი (Tkhili) | შდიხ (Shdikh Svan.) | (Khaka Svan.), (Nemsa Svan.) | Branches, Fruit, Leaf, Stem | Forest, Garden |
| **Boletaceae** |  |  |  |  |  |  |  |
| *Boletus edulis* Bull. | 65 | Food (Human food) | დათიკა სოკო, დათვის სოკო, ძირბუკა (Datik'a sok'o, Datvis soko, Dzirbuk'a) |  |  | Fruit | Forest |
| *Boletus erythropus* Pers. | 66 | Food (Human food) | წითელფეხა (Ts'itelpekha) | ხუშხუშა (Khushkhusha) |  | Fruit | Forest |
| **Boraginaceae** |  |  |  |  |  |  |  |
| *Symphytum caucasicum* M. Bieb. | 456 | Medicinal (Fractures, Furuncle, Gastro intestinal system) | ლაშქარა (Lashqara), შალდაყი (Shaldaq’i) | მელენ (Melen Svan.) |  | Leaf, Root | Forest, Garden |
| **Brassicaceae** |  |  |  |  |  |  |  |
| *Armeniaca vulgaris* Lam. | 38 | Food (Human food) | გარგარი (Gargari) |  |  | Fruit | Garden |
| *Armoracia rusticana* G. Gaertn., B. Mey. & Scherb. | 40 | Food (Human food) | პირშუშხა (P’irshushkha) |  |  | Leaf, Root | Garden |
| *Brassica campestris* L. | 70 | Food ((blank)) | შალგი (Shalgi) | გიერა (Giera Tush.) |  | Leaf | Garden |
| *Brassica campestris* L. ssp. *oleifera* DC. | 71 | Food (Human food, Phkhali, Pickled, Sats'ebai) | შალგი (Shalgi) | გიერა (Giera Tush.) |  | Leaf, Stem | Forest |
| *Brassica oleracea* L. | 72 | Food (Human food, Phkhali, Pickled) | კომბოსტო (K'ombost'o) | ლახანა (Lakhana Svan.) |  | Fruit, Leaf | Forest, Garden |
| *Brassica oleracea* L. Broccoli | 73 | Food (Human food, Pickled) | ბროკოლი (Brokoli) |  |  | Flower, Leaf | Garden |
| *Brassica oleracea* L. cauliflower | 74 | Food (Human food) | ყვავილოვანი კომბოსტო (Q'vavilovani k'ombost'o) |  |  | Flower, Leaf | Garden |
| *Brassica oleracea* L. red | 75 | Food (Human food) | ლურჯი კომბოსტო (Lurji k'ombost'o) |  |  | Leaf | Garden |
| *Brassica oleracea* L. var. *gemmifera* Brussles Sprouts | 76 | Food (Human food) | ბრიუსელის კომბოსტო (Briuselis k'ombost'o) |  |  | Leaf | Garden |
| *Brassica oleracea* L. var. *gongylodes* | 77 | Food (Human food) | კოლრაბი (K'olrabi) |  |  | Root | Garden |
| *Brassica rapa* L. subsp. *rapifera* Metzger | 78 | Food (Human food, Pickled) | თალგამი (Talgami), თალგამი (Talgami) | ქართ (Quart Svan.) |  | Fruit, Leaf, Root | Garden |
| *Brassica rapa* var. *rapa* L. | 79 | Food (Human food) | თალგამურა (Marts'q'vi), თალგამურა (Talgamura) |  |  | Root | Garden |
| *Bunias orientalis* L. | 80 | Food (Chave, Human food, Phkhali, Pickled); Medicinal (Against poisoning, Hangover, Parasites, Snakebite); Veterinary (Helminthes) | ხატოტი (Khat'ot'i), ხოხნუტა (Khotchadi) | გომატი (Gomat'i Tush.), ხოხნუტა (Khokhnuta Khev.) |  | Flower, Leaf, Leaf young, Seed, Stem | Forest |
| *Capsella bursa-pastoris* L. | 88 | Food (Human food, Phkhali) | წიწმატურა (Ts'ts'mat'ura), წიწმატურა (Ts’its’mat’ura), ხავარტა (Khavart’a) | ხავარტა (Khavart’a), ხარკბილა (Kharik'bila) |  | Leaf | Forest |
| *Cardamine hirsuta* L. | 91 | Food (Chachapuri, Phkhali) | ტყის წიწმატი (T'q'is ts'its'mat'i) |  |  | Leaf | Forest |
| *Cheiranthus cheiri* L. | 101 | Food (Phkhali) | შაბუ (Shabu) |  |  | Leaf | Forest |
| *Isatis tinctoria* L. | 271 | Utensils and tools (Dye) | საღებავი მათრახა (Saghebavi matrakha) |  |  | Leaf | Forest |
| Indet sp. 50 | 265 | Food (Chave, Human food, Phkhali, Sats'ebai) | ღორის ქადა (Ghoris qada) | ხარნუყა (Kharnuq’a Tush.) |  | Leaf | Forest |
| *Lepidium sativum* L. | 290 | Food (Human food) | წიწმატი (Ts'its'mat'i) |  |  | Leaf | Forest, Garden |
| *Raphanus sativus* L. var. *major* | 387 | Food (Human food) | ბოლოკი (Bolok’i), თალგამი (Talgami), მთის ბოლოკი (Mtis boloki), შავი ბოლოკი (Shavi Boloki) | მიწისმხალა (Mits'imkhala) |  | Leaf, Root | Garden |
| *Raphanus sativus* L. var. *major* black | 388 | Medicinal (Cold, Cough) | შავი ბოლოკი (Shavi Boloki) |  |  | Root | Garden |
| *Raphanus sativus* L. var. *major* white | 389 | Food (Human food) | თეთრი ბოლოკი (Tetri Boloki) |  |  | Root | Garden |
| *Raphinastrum rugosum* (L.) All. | 390 | Food (Phkhali) | ბოლოკა (Bolok'a), შალგი (Shalgi) |  |  | Leaf, Stem | Forest |
| *Sinapis arvensis* L. | 438 | Food (Human food, Phkhali, Pickled, Spice) | გიერა (Giera), მდოგვი (Mdogvi), მინდვრის მდოგვი (Marts'q'vi), მინდვრის მდოგვი (Mindvris mdogvi) |  |  | Leaf, Seed | Forest, Garden |
| **Bryophyta** |  |  |  |  |  |  |  |
| Ground Moss | 197 | Utensils and tools (Dye) | მიწის ხავსი (Mits’is khavsi) |  |  | Whole plant | Forest |
| **Campanulaceae** |  |  |  |  |  |  |  |
| *Campanula biebersteiniana* Roem. & Schult. | 83 | Food (Human food) | ქარცხვი (Kartskhvi) |  |  | Flower | Forest |
| *Campanula lactiflora* Bieb. | 84 | Food (Human food, Phkhali, Pickled) | კიცძიშლ (Kitsdzishl) | (Kitsdzishl Svan.), კენკეშა (K'enk'esha Khev.), ქიც (Kits Svan.) |  | Leaf, Stem | Forest |
| *Campanula rapunculoides* L. | 85 | Food (Human food, Sats'ebai) | მაჩიტა (Machit'a), მიჩიგტარაი (Michigt’arai) | მაჩიკა (Machika Khev.) |  | Leaf, Root | Forest |
| **Cannabaceae** |  |  |  |  |  |  |  |
| *Cannabis sativa* L. | 86 | Food (Chachapuri, Human food, Svan salt); Medicinal (Tumors); Utensils and tools (Rope) | კანაფი (K'anapi), ქან (Qan) |  |  | Seed, Stem | Garden |
| *Humulus lupulus* L. | 215 | Food (Beer) | სვე (Sve) |  |  | Flower | Forest |
| **Cantharellaceae** |  |  |  |  |  |  |  |
| *Cantharellus cibarius* Fr. | 87 | Food (Human food); Medicinal (Hepatitis, Liver) | მელაკუდა (Melakuda), მიქლიო (Miklio) | მიქუელა (Miquela Svan.) |  | Fruit, Leaf | Forest |
| **Caprifoliaceae** |  |  |  |  |  |  |  |
| *Lonicera caucasica* Pall. | 297 | Food (Human food); Medicinal (Heartburn); Utensils and tools (Arrows) | წერწა (Ts'erts'a) | ჭიჭკოტი (Ch’ich’k’ot’i Tush.) |  | Branches, Fruit, Leaf | Forest |
| **Caryophyllaceae** |  |  |  |  |  |  |  |
| *Melandrium balansae* Boiss. | 312 | Food (Phkhali); Medicinal | ვირთბატრა (Virtbat’ra), სასტვენა (Tsik’niq’ura) |  |  | Leaf | Forest |
| *Melandrium boissieri* Schischk. | 313 | Food (Phkhali); Medicinal; Utensils and tools (Whistles) | ვირთბატრა (Virtbat’ra), სასტვენა (Sastvena) | ბალანსა (Balansa Khev.), სასტვენა (Tsik’niq’ura) |  | Leaf, Stem | Forest |
| *Oberna wallichiana* (Klotzsch) Ikonn. | 327 | Food (phkhlovana) | ჭრიჭინა (Ch'ritchina) |  |  | Leaf | Forest |
| *Silene lacera* Steven | 436 | Food (Chachapuri, Chave, Human food, Khinkali, Phkhali) |  | ქვიშამხალი (KvishaPhkhali Tush.) |  | Leaf, Seed | Forest, Garden |
| *Silene wallachiana* Klotzsch | 437 | Food (Human food, Phkhali) | მჭივანა (Mch'ivana) |  |  | Leaf | Forest |
| **Cornaceae** |  |  |  |  |  |  |  |
| *Cornus australis* C.A. Mey. | 121 | Utensils and tools (Walking sticks) | შინდანწლა (Shvindants’la) |  |  | Stem | Forest |
| *Cornus mas* L. | 122 | Food (Human food); Utensils and tools (Barrel cleaner, Fighting sticks, Stirrer, Tool handles) | შვინდი (Shvindi), შინდი (Shindi) |  |  | Bark, Branches, Fruit, Stem | Forest, Garden |
| *Swida australis* (C.A. Mey.) Pojark ex Grossh. | 455 | Utensils and tools (Sieves) | შინდანწლა (Shindants'la) |  |  | Branches | Forest |
| **Cortinariaceae** |  |  |  |  |  |  |  |
| *Cortinarius violaceus* (L. ex Fr.) Gray | 124 | Food (Human food) | ლურჯკაბა (Lurjk'aba) |  |  | Fruit | Forest |
| **Crassulaceae** |  |  |  |  |  |  |  |
| *Sedum caucasicum* Boriss. | 432 | Food (Human food, Phkhali); Medicinal (Arthritis, Chaps, Corns, Furuncle, Rheumatism, Toothache, Wounds) |  | კლდის დუმა (K’ldisduma Tush.) |  | Leaf | Forest |
| *Sedum oppositifolium* Sims | 433 | Food (Human food, Phkhali); Medicinal (Furuncle, Toothache, Wounds) |  | კლდის დუმა (K’ldisduma Tush.) |  | Leaf | Forest |
| *Sempervivum caucasicum* Rupr. ex Boiss. | 434 | Food (Human food) | კლდისვაშლა (K'ldisvashla), კლდისვაშლა (Pkhija), ჯორისკუდა (Jorisk’uda) | კლდის დუმა (K’ldisduma Tush.) |  | Leaf | Forest |
| **Cucurbitaceae** |  |  |  |  |  |  |  |
| *Citrullus lanatus (*Thunb.) Matsum. & Nakai var. *lanatus* | 110 | Food (Human food, Pickled) | საზამთრო (Sazamtro) | (Dedinatsvlis ena) |  | Fruit | Garden |
| *Cucumis melo* L. | 130 | Food (Human food) | ნესვი (Nesvi) |  |  | Fruit | Garden |
| *Cucumis sativus* L. | 131 | Food (Human food, Pickled) | კიტრი (K'it'ri) |  |  | Flower, Fruit | Garden |
| *Cucurbita pepo* L. | 132 | Food (Human food, Phkhali, Pickled); Medicinal (Digestive system) | გოგრა (Gogra) | კვახი (Kvakhi Svan.), კობეშია (Kobeshia Svan.), ქუთკვახ (Qutkvakh Svan.) |  | Flower, Fruit, Leaf | Forest, Garden |
| *Cucurbita pepo* L. flat squash | 133 | Food (Human food) | გოგრა (gogra) |  |  | Fruit | Garden |
| *Cucurbita pepo* L. var. *giromontia* | 134 | Food (Human food) | ყაბაყი (Q'abaq'i) |  |  | Fruit | Garden |
| *Cucurbita pepo* L. var. *patisson* | 135 | Food (Human food) | ყაბაყი პატისონი (Q'abaq'i p'atisoni) |  |  | Fruit | Garden |
| *Cucurbita pepo* L. Zucchini | 136 | Food (Human food) | ყაბაყი (Q'abaq'i) |  |  | Flower, Fruit | Garden |
| **Cupressaceae** |  |  |  |  |  |  |  |
| *Juniperus depressa* Raf. ex M'Murtrie | 273 | Medicinal | ღვია (Ghvia) |  |  | Fruit | Forest |
| *Juniperus hemisphaerica* C.Presl | 274 | Medicinal (Blood cleansing, Kidneys, Urinary system) |  | წყერო (Tzqhero Svan.) |  | Branches | Forest |
| *Juniperus oblonga* Bieb. | 275 | Fuel (Firewood); Medicinal (Blood cleansing, Kidneys, Urinary system) | ღვია (Ghvia) | წყერო (Tzqhero Svan.) |  | Branches, Stem | Forest |
| *Junperus sabina* L. | 276 | Food (Human food); Medicinal (Blood cleansing, detoxicant, Diuretic, Eczema, Gallbladder, no se sabe, Prostate , Toothache, Urinary system); Veterinary (Diuretic, Skin problems) | ღვია (Ghvia) | (T'sveda Tush.), (Ts'q'veda), ჭყერო (Tchqhero Svan.) |  | Fruit, Leaf, Root, Stem | Forest |
| **Cystopteridaceae** |  |  |  |  |  |  |  |
| *Cystopteris fragilis* (L.) Bernh. | 139 | Medicinal (Wounds) | გვიმმრა (Gvimra) |  |  | Whole plant | Forest |
| **Dryopteridaceae** |  |  |  |  |  |  |  |
| *Dryopteris filix-mas* (L.) Schott. | 148 | Food (Human food, Phkhali, Pickled); Medicinal (Bruises) | ჩადუნა (Chaduna) | ჩადა (Chada Tush.) |  | Leaf | Forest |
| Indet sp. 47 | 262 | Medicinal (Burns) |  | დიდი იფხი (Didi iphkhi Svan.) |  | Root | Forest |
| *Mattheucia struthiopteris* (L.) Todd. | 311 | Food (Human food, Phkhali, Pickled) | გვიმრა (Gvimra), ჩადუნა (Chaduna) | Муча (Mucha Russ.), მუჩი (Muchi Svan.) |  | Leaf, Stem | Forest |
| **Ebenaceae** |  |  |  |  |  |  |  |
| *Diospyros lotus* L. | 146 | Food (Human food) | ჩვეულებრივი ხურმა (Khveulebrivi khurma) |  |  | Fruit | Forest |
| *Diospyros* sp. | 147 | Food (Human food) | ხურმა (Khurma) |  |  | Fruit | Garden |
| **Eleagnaceae** |  |  |  |  |  |  |  |
| *Hippophaë rhamnoides* L. | 211 | Medicinal (Diabetes, Vitamins) | ქაცვი (Katsvi) |  |  | Fruit | Forest, Garden |
| **Equisetaceae** |  |  |  |  |  |  |  |
| *Equisetum arvense* L. | 150 | Medicinal (Kidneys, Urinary system, Wounds) | შვიტა (Shvit'a) |  |  | Leaf, Stem | Forest |
| **Ericaceae** |  |  |  |  |  |  |  |
| *Empetrum hermaphroditum* Hagerup | 149 | Food (Human food); Utensils and tools (Brush to wash tools) | კეწერა (K'ets'era) |  |  | Branches, Fruit, Leaf | Forest |
| *Oxycoccus quadripetalus* Gilib. | 332 | Food (Human food) | შტოში (Shtoshi) |  |  | Fruit | Forest |
| *Vaccinium arctostaphylos* L. | 482 | Food (Alcohol, Human food, Tea, Wine); Medicinal (Anemia, Cold, Diabetes, Inflammation, Stomach) | მოცვი მაღალი (Motsvi maghali) | ცინყა (Tsinqha Svan.) |  | Fruit, Leaf | Forest, Garden |
| *Vaccinium myrtillus* L. | 483 | Food (Human food, Marmalade, Tea, Wine); Medicinal (Cold, Decreases blood sugar, Dry throat, Kidney stones, Tea); Utensils and tools (Dye) | მოცვი (Motsvi), ჟოლი (Zholi) | (Shalshavi Khev.), იღვი (ighvi Svan.), მეგმულდ (Megmuld Svan.), ჟოლი (Zholi Tush.), შელშავი (Shelshavi Khev.) |  | Branches, Flower, Fruit, Leaf | Forest |
| *Vaccinium vitis-idaea* L. | 484 | Food (Human food, Tea); Medicinal (Bedwetting in children, Decreases blood sugar, Liver); Utensils and tools (Dye) | წითელი მოცვი (Ts’iteli motsvi) | ვიღვი (Vighv Svan.), მაიოლ / მაია (Maiol / Maia Svan.), სტომი (Stomi Tush.), წითელმოჩა (Tsitelimocha Khev.) |  | Branches, Fruit, Leaf | Forest |
| **Fabaceae** |  |  |  |  |  |  |  |
| *Astragalus caucasisus* Pall. | 51 | Food (Tea) | გლერძი (Glerdzi) |  |  | Leaf | Forest |
| *Cicer arietinum* L. | 107 | Food (Human food) | მუხუდო (Mukhudo) |  |  | Seed | Garden |
| *Coronilla varia* L. | 123 | Food (Chachapuri) | ყვავისფრჩხილა (Q'vavisprchkhila) |  |  | Leaf | Forest |
| *Galega orientalis* Lam. | 191 | Food (Pickled) | ხბოშუბლა (Khboshubla) |  |  | Stem | Forest |
| *Glycine max* (L.) Merr. | 194 | Food (Human food) | მუხუდო (Mukhudo), სოია (Soia) | სოიო (Soio Svan.) |  | Leaf, Seed | Garden |
| *Glycyrrhiza glabra* L. | 195 | Food (Human food); Medicinal (Cough) | ძირტკბილა (Dzirt’k’bila) |  |  | Root | Forest |
| Indet sp. 48 | 263 | Medicinal | გლერძი (Glerdzi) |  |  | Latex | Forest |
| *Lathyrus roseus* Steven | 286 | Food (Phkhali) | ვაზისძირა (Vazisdzira) | არჯაკელი (Arjak'eli Tush.), ზერჩო (zercho Svan.) |  | Leaf | Forest |
| *Lens cornicularis* L. | 288 | Food (Human food) | ოსპი (Ospi) | ქირს (Qirs Svan.) |  | Seed | Garden |
| *Onobrychis viciifolia* Scop. | 329 | Animal food (Fodder) | ესპარცეტი (Esp'artseti) |  |  | Leaf | Garden |
| *Phaseolus sativus* L. | 338 | Food (Human food) | ლობიო (Lobio) |  |  | Fruit, Seed | Garden |
| *Phaseolus sativus* L. climbing variety | 339 | Food (Human food) | ლობიო (Lobio) |  |  | Fruit | Garden |
| *Phaseolus sativus* L. low variety | 340 | Food (Human food) | ლობიო (Lobio) |  |  | Fruit | Garden |
| *Phaseolus vulgaris* L. | 341 | Food (Human food) | ლობიო (Lobio) |  |  | Fruit, Seed | Garden |
| *Pisum sativum* L. | 348 | Food (Human food) | ბარდა (Barda), მუხუდო (Mukhudo) | ისაბ (Isab Svan.), ღედაარ (Ghedaar Svan.) |  | Fruit, Seed | Garden |
| *Robinia pseudoacacia* L. | 401 | Construction (Fences, Timber); Utensils and tools (Rope, Tool handles, Walking sticks) | აკაცია (Akatsia) |  |  | Bark, Stem | Forest, Garden |
| *Trifolium repens* L. | 471 | Animal food (Fodder) | სამყურა (Samq’ura) |  |  | Leaf | Garden |
| *Trifolium* sp. | 472 | Medicinal (Wounds); Utensils and tools (Dye) | სამყურა (Samq’ura) |  |  | Leaf | Forest |
| *Trigonella caerulea* (L.) Ser. | 473 | Food (Human food, Svan salt) | ულუმბო (Ulumbo), შამბრიკა (Shambrika) | უცხო სუნელი (Utskho suneli Svan.) |  | Leaf, Seed | Garden |
| *Vicia faba* L. | 490 | Food (Human food); Medicinal (Heartburn) | ცერცვი (Tsertsvi) | როგი (Rogv Svan.) |  | Fruit, Seed | Forest, Garden |
| *Vicia sativa* L. | 491 | Food (Sats'ebai) | ჭეკუნტელაი (Ch’ek’unt’elai) |  |  | Leaf | Garden |
| **Fagaceae** |  |  |  |  |  |  |  |
| *Castanea sativa* Mill. | 94 | Construction (Fences, Timber, Windows, Doors); Food (Human food); Utensils and tools (Carts, Tool handles, Walking sticks) | წაბლი (Tzabli) | გვიჯ (gvij Svan.) |  | Fruit, Stem | Forest, Garden |
| *Fagus orientalis* Lipsky | 154 | Construction (Timber); Food (Human food, Phkhali); Fuel (Firewood); Utensils and tools (Furniture, Sleds, Tool handles) | წიფელი (Ts'ipeli) | წიფრა (Tziphra Svan.) |  | Fruit, Leaf, Seed, Stem | Forest |
| *Quercus iberica* Steven ex M. Bieb. | 385 | Construction (Fences, Timber); Food (Human food); Fuel (Firewood); Utensils and tools (Barrels, Furniture, Tool handles); Veterinary (Diarrhea, Toothache) | მუხა (Mukha) | ჯიჰრა (Mihra Svan.) |  | Bark, Fruit, Juice, Leaf, Stem | Forest |
| **Fungi** |  |  |  |  |  |  |  |
| "Alnus barbata fungus" | 1 | Food (Human food) | მურყანისოკო (Murq’anisoko) |  |  | Fruit | Forest |
| Fungus sp. 1 | 166 | Food (Human food) | ჭრელკაბა (Ch'relk'aba) |  |  | Fruit | Forest |
| Fungus sp. 2 | 177 | Food (Human food) | (Gerda) |  |  | Fruit | Forest |
| Fungus sp. 3 | 183 | Food (Human food) | (Kruse) |  |  | Fruit | Forest |
| Fungus sp. 4 | 184 | Food (Human food) | ლარგი (Largi) |  |  | Fruit | Forest |
| Fungus sp. 5 | 185 | Food (Human food); Utensils and tools (Insecticide) | მარნულა (Marnula) |  |  | Fruit | Forest |
| Fungus sp. 6 | 186 | Food (Human food) | (Mindurisia) |  |  | Fruit | Forest |
| Fungus sp. 7 | 187 | Food (Human food) | (Mitisoko) |  |  | Fruit | Forest |
| Fungus sp. 8 | 188 | Food (Human food) | მიწის კალმახი (Mits'is k'almakhi) |  |  | Fruit | Forest |
| Fungus sp. 9 | 189 | Food (Human food) | მწარია (Mts'aria) |  |  | Fruit | Forest |
| Fungus sp. 10 | 167 | Food (Human food) | (Shantskava) |  |  | Fruit | Forest |
| Fungus sp. 11 | 168 | Food (Human food) | (Telasoko) |  |  | Fruit | Forest |
| Fungus sp. 12 | 169 | Food (Human food) | (Triasoko) |  |  | Fruit | Forest |
| Fungus sp. 13 | 170 | Food (Human food) | (Tianasoko) |  |  | Fruit | Forest |
| Fungus sp. 14 | 171 | Food (Human food) | (Tshadasoko) |  |  | Fruit | Garden |
| Fungus sp. 15 | 172 | Food (Human food) | (Tsiphela) |  |  | Fruit | Forest |
| Fungus sp. 16 | 173 | Food (Human food) | (Tsiphela) |  |  | Fruit | Forest |
| Fungus sp. 17 | 174 | Food (Human food) | (Tsirtselisoko) |  |  | Fruit | Forest |
| Fungus sp. 18 | 175 | Food (Human food) | (Tsvilisoko) |  |  | Fruit | Forest |
| Fungus sp. 19 | 176 | Food (Human food) | (Viteli) |  |  | Fruit | Forest |
| Fungus sp. 20 | 178 | Food (Human food) |  | Родственница (Rostvennitsa Russ.) |  | Fruit | Forest |
| Fungus sp. 21 | 179 | Food (Human food) |  | არჩექალი (Archekali Khev.) |  | Fruit | Forest |
| Fungus sp. 22 | 180 | Food (Human food) | (Telasoko) |  |  | Fruit | Forest |
| Fungus sp. 23 | 181 | Food (Human food) | (Telasoko) |  |  | Fruit | Forest |
| Fungus sp. 24 | 182 | Food (Human food) | (Telasoko) |  |  | Fruit | Forest |
| **Gentianaceae** |  |  |  |  |  |  |  |
| *Gentiana cruciata* L. | 192 | Medicinal (Gallbladder, Liver, Stomach) | ნაღველა (Naghvela), ნაღველა ჯვრისებრი (Naghvela jvrisebri) |  |  | Leaf | Forest |
| *Gentiana septemfida* Pall. | 193 | Medicinal (Cholagogic, Gallstones, Liver, Stomach) | ნაღველა (Naghvela) |  |  | Leaf | Forest |
| *Swertia iberica* Fisch & C.A. Mey. | 454 | Food (Chave) | გაბლუარაი (Gabluarai) |  |  | Leaf | Forest |
| **Gomphaceae** |  |  |  |  |  |  |  |
| *Ramaria flava* (Schaeff.) Quél. | 386 | Food (Human food) | საჩეჩელა (Sachechela) | საჩიჩელა (Sachichela Svan.), ბაწარა (Bats'ara) |  | Fruit | Forest |
| **Grossulariaceae** |  |  |  |  |  |  |  |
| *Grossularia reclinata* (L.) Mill. | 196 | Food (Human food) | ხურტკმელი (Khurt’k’meli) |  |  | Fruit | Forest, Garden |
| *Ribes biebersteinii* Berl. ex DC | 394 | Food (Human food, Tea) | მოცხარი (Motskhari) | მენცხვარი (Mantskhald Svan.), ხუნწი (Khunts'i Tush.) |  | Fruit, Leaf | Forest, Garden |
| *Ribes grossularia* L. | 395 | Food (Human food) |  | ოფლეენდ (Ophleend Svan.) |  | Fruit | Forest, Garden |
| *Ribes nigrum* L. | 396 | Food (Human food) | მოცხარი (Marts'q'vi), მოცხარი (Motskhari), შავი მოცხარი (Shavi motskhari) |  |  | Fruit | Forest, Garden |
| *Ribes orientale* Desf. | 397 | Food (Human food) | ალუდა (Aluda) |  |  | Fruit | Forest |
| *Ribes rubrum* L. | 398 | Food (Human food) | მოცხარი (Motskhari) |  | (red) | Fruit | Garden |
| *Ribes uva-crispa* L. | 399 | Food (Human food) | ხურტკმელი (Khurt’k’meli) | ოფლანდ (Ophleend Svan.) |  | Fruit | Forest, Garden |
| *Ribes vulgare* Lam. | 400 | Food (Human food) | წითელი მოცხარი / ალუდა (Ts’iteli motskhari) |  |  | Leaf | Forest |
| **Hericiaceae** |  |  |  |  |  |  |  |
| *Hericium erinaceus* (Bull.) Pers. | 209 | Food (Human food) | ეშმაკის ბურნუთი (Eshmak'is burnuti) | გუდასოკო (Gulasoko Tush.) |  | Fruit | Forest |
| **Hypericaceae** |  |  |  |  |  |  |  |
| *Hypericum perforatum* L. | 218 | Cultural (Perfume); Food (Beer, Tea); Medicinal (Enuresis, Gallbladder, Gums, Kidneys, Liver, Nerves, Oral inflammation, Panacea, Ulcers); Utensils and tools (Dye) | კრაზანა (K’razana) |  |  | Branches, Flower, Leaf, Whole planta | Forest, Garden |
| **Indeterminatus** |  |  |  |  |  |  |  |
| Indet sp. 1 | 220 | Food (Human food) | (Acara) |  |  | Fruit | Garden |
| Indet sp. 2 | 232 | Food (Human food) | (Barishindi) |  |  | Fruit | Garden |
| Indet sp. 3 | 243 | Food (Human food) | (Brasidz) |  |  | Fruit | Garden |
| Indet sp. 4 | 254 | Food (Chachapuri) | (Ch'ailova) |  |  | Leaf | Forest |
| Indet sp. 5 | 264 | - | (Ch'ardli) |  |  | Leaf | Forest |
| Indet sp. 6 | 266 | Food (Phkhali) | (Ch'areshi) |  |  | Leaf | Forest |
| Indet sp. 7 | 267 | Food (Human food) | (Ch'erani) |  |  | Fruit | Garden |
| Indet sp. 8 | 268 | Food (Chachapuri) | (Ch'ichilag) |  |  | Leaf | Forest |
| Indet sp. 9 | 269 | Food (Human food) | (Ikha) |  |  | Fruit | Garden |
| Indet sp. 10 | 221 | Food (Human food) | (Jatami) |  |  | Fruit | Garden |
| Indet sp. 11 | 222 | Food (Human food) | (Kareuli Chichmati) |  |  | Fruit | Forest |
| Indet sp. 12 | 223 | Food (Chachapuri) | (Khotshlivasha) |  |  | Leaf | Forest |
| Indet sp. 13 | 224 | Utensils and tools (Tool handles) | მლჯარა (M'ljara) |  |  | Stem | Forest |
| Indet sp. 14 | 225 | Food (Human food) | (Mitsichala) |  |  | Fruit | Garden |
| Indet sp. 15a | 226 | Food (Human food) | (Mushmula) |  |  | Fruit | Garden |
| Indet sp. 15b | 227 | Food (Human food) | (Mtsvane Maghvali) |  |  | Leaf | Garden |
| Indet sp. 16 | 228 | Food (Human food) | (Negoshali) |  |  | Leaf | Forest |
| Indet sp. 17 | 229 | - | (Nivris) |  |  | Leaf | Forest |
| Indet sp. 18 | 230 | - | (Potolishelva) |  |  | Leaf | Forest |
| Indet sp. 19 | 231 | Food (Human food) | (Sakrana) |  |  | Leaf | Garden |
| Indet sp. 20 | 233 | Food (Human food) | შავი (Shavi) |  |  | Fruit | Forest |
| Indet sp. 21 | 234 | Food (Human food) | (Soditz) |  |  | Leaf | Forest |
| Indet sp. 22 | 235 | - | (Tuji) |  |  | Leaf | Forest |
| Indet sp. 23 | 236 | Food (Human food) | (Ts'ahui) |  |  | Fruit | Garden |
| Indet sp. 24 | 237 | Medicinal (Liver) | (Tsavik) | (Antaran Arm.) |  | Leaf | Forest |
| Indet sp. 25 | 238 | Food (Human food) | (Tvili) |  |  | Fruit | Forest |
| Indet sp. 26 | 239 | - | (Zhila) |  |  | Leaf | Forest |
| Indet sp. 27 | 240 | Food (Human food) |  | (Achali Arm.) |  | Fruit | Forest |
| Indet sp. 28 | 241 | Medicinal (Liver) |  | (Antaran Arm.) |  | Leaf | Forest |
| Indet sp. 29 | 242 | Food (Human food) |  | (Dachit Arm.) |  | Leaf | Forest |
| Indet sp. 30 | 244 | Food (Human food) |  | დედოფალა (Dedophala Svan.) |  | Fruit | Forest |
| Indet sp. 31 | 245 | Food (Human food) |  | ჰარდლი (Haardl Svan.) |  | Stem | Forest |
| Indet sp. 32 | 246 | Food (Phkhali) |  | ჰაინერ (Hainer Svan.) |  | Leaf | Forest |
| Indet sp. 33 | 247 | Food (Phkhali) |  | (Jochola Khev.) |  | Leaf | Forest |
| Indet sp. 34 | 248 | Medicinal (Pankreatitis) |  | (Kalasjoti Arm.) |  | Leaf | Forest |
| Indet sp. 35 | 249 | Food (Human food) |  | (Muraba Russ.) |  | Fruit | Forest |
| Indet sp. 36 | 250 | Food (Phkhali) |  | ნესგვლა (Nesgvla Svan.) |  | Leaf | Forest |
| Indet sp. 37 | 251 | Food (Human food) |  | (Sindz Arm.) |  | Fruit | Forest |
| Indet sp. 38 | 252 | Food (Pickled) |  | (Tatjanura Arm.) |  | Stem | Forest |
| Indet sp. 39 | 253 | Food (Human food) |  | (Teterjik Arm.) |  | Fruit | Forest |
| Indet sp. 40 | 255 | Utensils and tools (Furniture) |  | ცხალი (Tskhali Svan.) |  | Stem | Forest |
| Indet sp. 41 | 256 | Food (Phkhali) |  | ცუჟლა (Tzushla Svan.) |  | Leaf | Forest |
| Indet sp. 42 | 257 | Food (Human food) |  | (Uremi Arm.) |  | Stem | Forest |
| Indet sp. 43 | 258 | Food (Human food) |  | ვერდელი (Verdel Svan.) |  | Stem | Garden |
| Indet sp. 44 | 259 | Food (Human food) |  | (Vertshik Arm.) |  | Fruit | Forest |
| Indet sp. 45 | 260 | Food (Pickled) |  | ვირდუცა (Virdutsa Khev.) |  | Stem | Forest |
| Indet sp. 46 | 261 | Food (Phkhali) |  | (Zestrula Khev.) |  | Leaf | Forest |
| **Juglandaceae** |  |  |  |  |  |  |  |
| *Juglans regia* L. | 272 | Construction (Timber); Food (Human food, Phkhali, Svan salt, Tea); Utensils and tools (Dye, Furniture, Household utensils) | ნიგოზი (Nigozi) | კაკალი (Kakali Svan.) |  | Fruit, Seed, Stem | Forest, Garden |
| *Pterocarya pterocarpa* (Michx.) Kunth ex Iljinsk. | 378 | Utensils and tools (Dye) | ლაფანი (Lapani) |  |  | Leaf | Forest |
| **Lamiaceae** |  |  |  |  |  |  |  |
| *Clinopodium vulgare* L. | 116 | Medicinal (Hypertension) | მოპიტნაო (Mopit'nao) |  |  | Leaf | Forest |
| *Lamium album* L. | 283 | Food (Human food, Phkhali); Medicinal (Bath for small children, Hair loss) | ჭინჭრის-დედა (Ch’inch’ris-deda) | Chinceris deda |  | Leaf, Whole plant | Forest |
| *Leonurus quinquelobatus* Gilib. var. *caucasicus* Krestovsk. | 289 | Medicinal (Epilepsy, Heart) | შავბალახა (Shavbalakha) |  |  | Leaf | Forest |
| *Melissa officinalis* L. | 314 | Medicinal (Nerves) | ბარამბო (Barambo) |  |  | Leaf | Forest |
| *Mentha aquatica* L. | 315 | Food (Spice) | პიტნა (Pit’na) |  |  | Leaf | Forest |
| *Mentha longifolia* (L.) L. | 316 | Food (Chave, Human food, Phkhali, Tea); Medicinal (Nerves, Panacea, Tea) | ტყის პიტნა (T'q'is p'it'na) | შანტალი პიტნაი (Shant’ali pitn'a Tush.) |  | Flower, Fruit, Leaf | Forest, Garden |
| *Mentha pulegium* L. | 317 | Food (Cheese suluguni, Human food, Tea) | ომბალო (Ombalo), პიტნა (Pit’na) |  |  | Fruit, Leaf | Garden |
| *Mentha* x *piperita* L. | 318 | Food (Human food, Phkhali, Pickled, Svan salt, Tea); Medicinal (Nerves, Panacea) | ბაღის პიტნა (Baghis pit'na) |  |  | Flower, Leaf | Forest, Garden |
| *Nepeta mussinii* Spreng. | 324 | Food (Tea) | პიტნა (Pit’na) |  |  | Leaf | Forest |
| *Ocimum basilicum* L. | 328 | Food (Human food, Svan salt) | რეჰანი (Rehani), შაშკულავი (Shashk'ulavi) |  |  | Fruit, Leaf | Garden |
| *Origanum vulgare* L. | 330 | Food (Beer, Human food, Spice, Tea); Medicinal (Lungs, Tea); Utensils and tools (Dye) | თავშავა (Tavshava) | душица (Dushitza Russ.) |  | Leaf | Forest, Garden |
| *Salvia nemorosa* L. | 423 | Medicinal (Enuresis) | დაჯირა (Dajira) |  |  | Leaf | Forest |
| *Salvia verticillata* L. | 424 | Medicinal (Anti-inflammatory, Enuresis, Wounds); Utensils and tools (Filter) | დაჯირა (Dajira) |  |  | Leaf, Whole plant | Forest, Garden |
| *Satureja hortensis* L. | 428 | Food (Human food, Svan salt, Tea) | ქონდარი (Kondari) |  |  | Leaf | Forest, Garden |
| Satureja laxiflora K. Koch | 429 | Food (Human food) | მინდვრის ქონდარი (Mindvris kondari) |  |  | Leaf | Forest |
| *Satureja spicigera* Boiss. | 430 | Food (Svan salt, Tea); Medicinal (Diabetes) |  | ჭვინ (Tchvin Svan.) |  | Leaf | Forest |
| *Thymus caucasicus* Willd. ex Benth | 461 | Food (Tea) | ქონდარი (Kondari), ბეგქონდარა (Begkondara) |  |  | Branches, Flower, Leaf | Forest |
| *Thymus colinus* Bieb. | 462 | Food (Spice, Tea); Medicinal (Cough, Hypertension) | ბეგქონდარა (Begkondara), ქონდარი (Kondari) |  |  | Leaf | Forest |
| *Thymus* sp. | 463 | Food (Human food, Tea) | ქონდარი (Kondari) | (Zetroni Arm.) |  | Fruit, Leaf | Forest, Garden |
| *Thymus transcaucasicus* Ronninger | 464 | Food (Human food, Spice) | ბეგქონდარა (Begkondara) | (Bektkondara Tush.), (Zetroni Arm.) |  | Leaf | Forest |
| *Ziziphora pushkinii* Adams. | 497 | Food (Tea); Medicinal (Antibiotic, Diuretic, Hypertension) | ურცი (Urtsi), ქონდარი (Kondari) | ბეგქონდარა (Bektkondara Tush.) |  | Leaf | Forest |
| *Ziziphora serpyllacea* M. Bieb. | 498 | Food (Spice, Tea) | ურცი (Urtsi) | ბეგქონდარა (Begkondara Tush.) |  | Leaf | Forest |
| **Lauraceae** |  |  |  |  |  |  |  |
| *Laurus nobilis* L. | 287 | Food (Human food); Utensils and tools (Dye) | დაფნა (Dapna) |  |  | Fruit, Leaf | Forest, Garden |
| **Lepiotaceae** |  |  |  |  |  |  |  |
| *Macrolepiota procera* (Scop.) Springer | 302 | Food (Human food) | წერეწო (Ts'erets'o), წეროსწვივა (Ts'erosts'viva) | ხარხუშა (Kharkhusha), ხუშხუშა (Khushkhusha) |  | Fruit | Forest |
| **Lichenes** |  |  |  |  |  |  |  |
| Rock Lichen | 402 | Utensils and tools (Dye) | კლდის ხავსი / ჯანგარო (K’ldis khavsi / Jangaro) |  |  | Whole plant | Forest |
| **Liliaceae** |  |  |  |  |  |  |  |
| *Fritillaria lutea* Mill. | 165 | Food (Human food); Medicinal (Hangover, Heart) | ყვითელი ღვინა (Q'viteli ghvina) | დათვკიტრა (Datvk’it’ra Khev.), მთის კიტრა (Mtis k'itra Kh,) |  | Bulb, Flower | Forest |
| *Galanthus* sp. | 190 | Food (Human food) | თეთრყვავილა (Tetrq'vavila) |  |  | Bulb | Forest |
| *Lilium szovitsianum* Fisch. & Avé-Lall. | 295 | Medicinal | დათვისკიტრა (Datvisk’it’ra) |  |  | Bulb | Forest |
| *Polygonatum glaberrimum* C. Koch. | 353 | Food (Chave) | სვინტრაი (Svintrai) |  |  | Leaf | Forest |
| *Veratrum lobelianum* Bernh. | 486 | Medicinal (Ektoparasites); Veterinary (Ektoparasites, Wounds) | შხამა (Shkhama) |  |  | Leaf, Root, Stem | Forest |
| **Linaceae** |  |  |  |  |  |  |  |
| *Linum usitatissimum* L. | 296 | Food (Human food); Medicinal (Trauma) | ქუმელი (Kumeli) | სელის ქუმელი (Selis qumeli Khev.) |  | Seed | Forest, Garden |
| **Lythraceae** |  |  |  |  |  |  |  |
| *Punica granatum* L. | 379 | Food (Human food) | ბროწეული (Brots'euli) |  |  | Fruit | Garden |
| **Malvaceae** |  |  |  |  |  |  |  |
| *Malva neglecta* L. | 307 | Food (Chachapuri, Human food, Phkhali) | ბალბა (Balba) | (Keji Arm.) |  | Fruit, Leaf | Forest, Garden |
| *Malva sylvestris* L. | 308 | Food (Chachapuri, Human food, Phkhali) | ბალბა (Balba) | (Keji Arm.) |  | Fruit, Leaf | Forest, Garden |
| *Tilia caucasica* Rupr. | 466 | Construction (Timber); Cultural (Musical instruments); Food (Tea); Utensils and tools (Household utensils, Tool handles, Vessels for pickling) | ცაცხვი (Phakha), ცაცხვი (Tsatskhvi) | ფაცაირიმა (Phatsaraima Svan.) |  | Flower, Stem | Forest |
| *Tilia begonifolia* Stev. | 465 | Construction (Timber); Cultural (Smoking); Food (Tea); Medicinal (Cold, Tea); Utensils and tools (Furniture, Household utensils, Rope) | ცაცხვი (Phakha), ცაცხვი (Tsatskhvi) |  |  | Bark, Flower, Fruit, Leaf, Stem | Forest, Garden |
| *Tilia cordata* Mill. | 467 | Utensils and tools (Household utensils) | ცაცხვი (Phakha) |  |  | Stem | Forest |
| **Marasmiaceae** |  |  |  |  |  |  |  |
| *Marasmius oreades* (Bolton) Fr. | 309 | Food (Human food) | წრიალა (Ts'riala) |  |  | Fruit | Forest |
| **Moraceae** |  |  |  |  |  |  |  |
| *Ficus carica* L. | 157 | Food (Human food) | ლეღვი (Leghvi) |  | (black), (white) | Fruit | Garden |
| *Morus alba* L. | 322 | Food (Alcohol, Human food, Marmalade); Utensils and tools (Tool handles) | თუთა (Tuta) |  | (black), (white) | Fruit, Stem | Forest, Garden |
| **Morchellaceae** |  |  |  |  |  |  |  |
| *Morchella conica* Pers | 320 | Food (Human food) | ხარისფაშვა (Kharispashva) |  |  | Fruit | Forest |
| *Morchella escuenta* Fr. | 321 | Food (Human food) | ხარისფაშვა (Kharispashva) |  |  | Fruit | Forest |
| **Musaceae** |  |  |  |  |  |  |  |
| *Musa* x *paradisiaca* L. | 323 | Ornamental (Ornamental) | ბანანი (Banani) |  |  | Whole plant | Garden |
| **Oleaceae** |  |  |  |  |  |  |  |
| *Fraxinus excelsior* L. | 164 | Construction (Timber); Food (Human food); Utensils and tools (Sleds, Tool handles) | იფანი (Ipani) | ლაჯრა (lajra Svan.), წითელა (Mzitela Svan.) |  | Leaf, Stem | Forest |
| **Onagraceae** |  |  |  |  |  |  |  |
| *Chamenaerion angustifolium* (L.) Holub. | 100 | Food (Chachapuri); Medicinal (Tea) | თხაწართხალა (Mhkhatz'artkhala) | Иван-чай (Ivantshai Russ.) |  | Leaf | Forest |
| **Ophioglossaceae** |  |  |  |  |  |  |  |
| *Botrychium lunaria* (L.) Sw. | 67 | Medicinal (Panacea, Wounds) | მარგალიტა (Margalit'a), წყლულის ბალახი (Ts’q’lulis balakhi) |  |  | Leaf, Whole plant | Forest |
| **Oxalidaceae** |  |  |  |  |  |  |  |
| *Oxalis acetosela* L. | 331 | Food (Human food) | მჟაველა (Mzhavela) |  |  | Leaf | Forest |
| **Papaveraceae** |  |  |  |  |  |  |  |
| *Chelidonium majus* L. | 102 | Medicinal (Panacea, Warts, Wounds); Utensils and tools (Dye) | ქრისტესისხლა (Kristesiskhla) |  |  | Latex, Leaf | Forest |
| **Parmeliaceae** |  |  |  |  |  |  |  |
| *Usnea* sp. | 481 | Utensils and tools (Dye) | ირმის ხავსი (Irmis khavsi) |  |  | Whole plant | Forest |
| *Usnea barbata* | 480 | Utensils and tools (Dye) | ბაღლაწო (Baghlats’o) |  |  | Whole plant | Forest |
| **Physalacriaceae** |  |  |  |  |  |  |  |
| *Armillariella mellea* (Vahl) P. Kumm | 39 | Food (Human food) | მანჭკვალა (Mantchkvala) |  |  | Fruit | Forest |
| **Pinaceae** |  |  |  |  |  |  |  |
| *Abies nordmanniana (*Steven) Spach | 2 | Construction (Timber); Food (Human food, Phkhali, Tea); Medicinal (Tuberculosis, Ulcers); Utensils and tools (Sleds) | სოჭი (Sotchi) | ჭიშხ (Tshishkh Svan.) |  | Branches, leaf, Stem | Forest |
| *Cedrus* sp. | 95 | Food (Human food) | კედარი (Kedari) |  |  | (blank) | Garden |
| *Picea orientalis* (L.) Peterm. | 344 | Construction (Roof shingles, Timber); Food (Human food, Masticant); Medicinal (Bronchitis, Tuberculosis, Ulcers, Wounds); Utensils and tools (Sleds, Tool handles, Walking sticks) | ნაძვი (Nadzvi) | ხაარ (Khaar Svan.) |  | Leaf, Pollen, Resin, Stem | Forest |
| *Pinus kochiana* Klotzsch ex K. Koch | 345 | Construction (Posts, Roof shingles, Timber); Cultural (Masticant); Food (Human food, Sweets); Fuel (Firestarter, Firewood); Medicinal (Allergies, Asthma, Bronchitis, Burns, Cough, Diuretic, Fungal diseases, Kidneys, Lungs, Sinusitis, Tuberculosis, Ulcers); Utensils and tools (Arrows, Bows, Boxes, Furniture, Grain storage trunks, Household utensils, Lanterns, Loom, Shelves, Sleds, Spinning wheels, Tool handles, Trunks) | ბუკვანი (Buk’vani), ფიჭვი (Phich’vi), ფიჭვის გირჩები (Pich'vis girchebi), ხალცუცა (Khaltsutsa) |  |  | Bark, Branches, Cones, Fruit, Leaf, Oil, Pollen, Resin, Root, Stem | Forest |
| **Plantaginaceae** |  |  |  |  |  |  |  |
| *Plantago major* L. | 349 | Medicinal (Bleeding, Cough, Digestive system, Gastritis, Gastro intestinal system, Intestines, Stomach, Wounds) | მრავალძარღვა (Mravaldzarghva) | (Tsxradzarghva Tush.), კუთკვახ (Kutkvakh Svan.), ცხრაძარღვა (Tkhradzargva Khev.), ჯოოდიბალე (Jo’odibale Svan.) |  | Latex, Leaf, Root | Forest, Garden |
| **Pleurotaceae** |  |  |  |  |  |  |  |
| *Pleurotus cornicopiae* (Paulet) Rolland | 350 | Food (Human food) | მაღვალი (Maghvali), მაჩალოსოკო (Machalosoko) |  |  | Fruit | Forest |
| *Pleurotus ostreatus* (Jacq. ex Fr.) P. Kumm | 351 | Food (Human food) | კალმახა (K'almakha), ხის სოკო (Khis soko) | (Tsiplis soko), ტყუბულ (Tqhubul Svan.) |  | Fruit | Forest, Garden |
| **Pluteaceae** |  |  |  |  |  |  |  |
| *Pluteus cervinis* (Schaeffer ex Fr.) P. Kumm. | 352 | Food (Human food) | ირმის რქა (Irmis rqa) |  |  | Fruit | Forest |
| **Poaceae** |  |  |  |  |  |  |  |
| *Avena sativa* L. | 54 | Food (Human food) | შვრია (Shvira) | ზინთხ (Zintkh Svan.) |  | Seed | Garden |
| *Festuca djimilensis* Boiss. & Balansa | 156 | Utensils and tools (Stuffing for shoes) |  | წერექვ (Tzereqv Svan.) |  | Leaf | Forest |
| *Hordeum violaceum* Boiss. & Huet | 212 | Medicinal (Cancer) | ქერი (Keri) |  |  | Leaf | Forest |
| *Hordeum vulgare* L. | 213 | Food (Alcohol, Beer, Human food) | მუხუდო (Mukhudo), ორრიგა (Orriga keri), ქერი (Keri) | ჭმინ (Tchmin Svan.) | (Qershveli) | Seed | Forest, Garden |
| *Hordeum vulgare* L. ssp. *vulgare* L. var. *coelestre* L. | 214 | Food (Human food) | ქერშველი (Kershveli) |  |  | Seed | Garden |
| *Panicum milanjianum* Rendle | 334 | Food (Human food) | ფეტვი (Phatvi) | წვინი (Tsvini Svan.) | (black), (white), (yellow) | Seed | Garden |
| *Phleum pratense* L. | 342 | Animal food (Fodder) | ტიმოთელა (T'imotela) |  |  | Leaf | Garden |
| *Secale cereale* L. | 431 | Food (Alcohol, Beer, Human food); Medicinal (Cold, Flu) | მუხუდო (Mukhudo), ჭვავი (Ch’vavi) | მანააშ (Manaash Svan.) | ორმწკრივი (Ormts’k’rivi), ქერშველა (Kershvela) | Seed | Garden |
| *Triticum aestivum* L. | 474 | Food (Alcohol, Beer, Human food) | ხორბალი (Khorbali) |  | (makha), (Mukhudo), (Uk’bilo puri), (zanduri), იფქლი (ipkli), კვეცერ (summer kvetser), კულ (winter kul) | Fruit, Seed | Garden |
| *Triticum carthlicum* Nevski | 475 | Food (Human food) | დიკა (Dika) |  |  | Seed | Garden |
| *Zea mays* L. | 496 | Food (Human food) | სიმინდი (Simindi) |  | (black), (summer), (white), (yellow) | Seed | Garden |
| **Polygonaceae** |  |  |  |  |  |  |  |
| *Bistorta officinalis* Delarbre | 64 | Medicinal (Diarrhea, Lungs) | მატიტელა (Matitela) | ჭიჭიშვილი (Tchitchishvili Khev.) |  | Flower, Root | Forest |
| *Fagopyrum tataricum* (L.) Gaertn. | 153 | Food (Human food) | წიწიბურა (Ts'its'ibura) |  |  | Seed | Garden |
| *Persicaria maculosa* Gray | 335 | Medicinal ((blank)) | მატიკელა (Matikela) |  |  | Leaf | Forest |
| *Polygonum alpinum* All. | 354 | Food (Chachapuri, Human food, Khinkali, Phkhali, Pickled, Sats'ebai); Medicinal (Gastro intestinal system, Joints); Utensils and tools (Dye) | წართხალი (Ts’artkhali) | ლეცირ (Letsir Svan.), ჭიჭიშვილი (Tsets'ich'ala Khev.) |  | Fruit, Leaf, Root, Stem | Forest, Garden |
| *Polygonum aviculare* L. | 355 | Food (Human food); Medicinal (Bladder, Diuretic, Kidneys, Urinary system) | მატიტელა (Matitela) | ჭიმჭიკი (Ch’imch’ik’I Tush.) |  | Leaf | Forest |
| *Polygonum carneum* C. Koch | 356 | Medicinal (Cirrhosis, Diarrhea, Liver, Lungs) | დვალურა (Dvalura), მატიტელა (Matitela) | ჭიჭიშვილი (Tchitchishvili Khev.) |  | Flower, Leaf, Root | Forest, Garden |
| *Polygonum hydropiper* L. | 357 | Medicinal Bruises, Fractures) | ჩაღანდრი (Chaghandri) |  |  | Leaf, Whole plant | Forest |
| *Polygonum* sp. | 358 | Food (Human food, Phkhali) | მამლაყინწა (Mamlaq’ints’a) |  |  | Leaf | Forest |
| *Rumex acetosa* L. | 411 | Food (Chachapuri, Human food, Phkhali, Pickled, Sats'ebai) | მჟაუნა (Mzhauna), მწყემსთმჟავიაი (Mts’q’emst mzhaviai), ყანის მჟავიაი (Q’anis mzhaviai) | (Teterdjik Arm.), (Dakht Arm.), ტელეფ (Teleph Svan.) |  | Leaf, Stem | Forest, Garden |
| *Rumex acetosella* L. | 412 | Food (Chachapuri, Human food, Phkhali, Pickled) | მჟაუნა (Mzhauna) |  |  | Leaf | Forest |
| *Rumex alpinus* L. | 413 | Food (Chave, Phkhali, Pickled); Medicinal (Colitis, Haemorrhoids, Intestines, Swellings, Tumors); Utensils and tools (Dye) | ღოლო (Gholo) | (Kvalo Arm.), საგუგა (Saguga Khev.), ჭირთალი (Ch'irtali Khev.), ჭირტალი (Ch’irt’ali Tush.) |  | Leaf, Root, Seed, Stem | Forest |
| *Rumex crispus* L. | 414 | Food (Chave, Human food, Phkhali, Pickled); Medicinal (Diarrhea); Utensils and tools (Dye) | ღოლო (Gholo) | (Avelug Arm.) |  | Leaf, Root, Seed, Stem | Forest |
| *Rumex scutatus* L. | 415 | Food (Human food, Phkhali, Pickled) | ლახტარა (Lakht'ara), ქვიშის მჟავია (Kvishis mzhavia) |  |  | Leaf, Stem | Forest |
| *Rumex tuberosu*s L. | 416 | Food (Spice) | მჟაუნა (Mzhavia) |  |  | Leaf | Forest |
| *Spinaca oleracea* L. | 449 | Food (Human food) | ისპანახი (Isp'anakhi) |  |  | Leaf | Garden |
| **Polypodiaceae** |  |  |  |  |  |  |  |
| *Polypodium vulgare* L. | 359 | Food (Human food, Sugar); Medicinal (Cough) | კილამურა (K’ilamura), ძირტკბილა (Dzirt’k’bila) |  |  | Root | Forest |
| **Polyporaceae** |  |  |  |  |  |  |  |
| *Piptoporus betulinus* (Bull.) P. Karst. | 346 | Food (Human food); Medicinal (Cancer) | ჩაგა (Chaga) |  |  | Fruit | Forest |
| *Polyporus squamosus* (Huds.) Fr. | 360 | Food (Human food) | ძერანა (Dzerana) |  |  | Fruit | Forest |
| **Portulacaceae** |  |  |  |  |  |  |  |
| *Portulaca oleracea* L. | 362 | Food (Human food, Phkhali) | დანდური (Danduri) |  |  | Leaf | Forest |
| **Primulaceae** |  |  |  |  |  |  |  |
| *Cyclamen vernum* Sweet | 137 | Food (Pickled) | ყოჩივარდა (Q'ochivarda) |  |  | Root | Forest |
| *Primula luteola* Rupr. | 363 | Food (Sats'ebai) |  | ვაშლისულა (Vashlisula Tush.) |  | Leaf | Forest |
| *Primula macrocalyx* Bunge | 364 | Food (Chave, Human food, Phkhali); Medicinal (Barrenness, Cough, Kidneys) | ფურისულა (Phurisula) | ვაშლისულა (Vashlisula Tush.) |  | Flower, Leaf | Forest |
| *Primula woronowii* Losinsk. | 365 | Food (Phkhali) | ტყის ფურისულა (T'q'is purisula) | ვაშლისულა (Vashlisula Tush.) |  | Leaf | Forest |
| **Psathyrellaceae** |  |  |  |  |  |  |  |
| *Coprinopsis atramentaria* (Bull.) Redhead, Vilgalys & Moncalvo | 118 | Food (Human food) | მელანა (Melana), მელანა, სილიო (Melana sok'o, silio) |  |  | Fruit | Forest |
| **Ranunculaceae** |  |  |  |  |  |  |  |
| *Aruncus vulgaris* Raf. | 48 | Food (Human food, Phkhali, Pickled) | (Marts'q'vi), მეკენძალა (Mek'endzala) | მეჭეხი (Metchekhi Svan.) |  | Leaf, Stem | Forest |
| *Clematis vitalba* L. | 115 | Food (Phkhali) |  | ციცაბალბა (Tsitsabalba Svan.) |  | Branches | Forest |
| *Helleborus caucasicus* R. Br. | 201 | Medicinal (Sinusitis); Veterinary (Bad liquid, Gas, Wounds) | ხარისძირა (Kharisdzira) |  |  | Root | Forest |
| **Rhododendraceae** |  |  |  |  |  |  |  |
| *Rhododendron caucasicum* Pall. | 392 | Food (Beer, Dye, Human food, Phkhali, Sats'ebai, Tea); Medicinal (Anti-inflammatory, Cold, Digestive system, Diuretic, Heart, Intestines, Lowers potency, Tea); Utensils and tools (Dye) | დეკა (Dek’a), წითელა (Ts'itela), წითელი (Ts’iteli) | შქერი (Shgver Svan.) |  | Branches, Flower, Fruit, Leaf | Forest, Garden |
| *Rhododendron luteum* Sweet | 391 | Construction (Roof support); Poison (Toxic) | იელი (Ieli) |  |  | Branches, Leaf, Whole plant | Forest, Garden |
| *Rhododendron ponticum* L. | 393 | Food (Tea) |  | შქერი (Shgver Svan.) |  | Leaf | Forest |
| **Rosaceae** |  |  |  |  |  |  |  |
| *Cotoneaster multiflorus* Bunge | 127 | Food (Human food) | ვაშლანა (Vashlana) |  |  | Fruit | Forest |
| *Crataegus curvisepala* Lindm. | 128 | Food (Human food, Tea); Medicinal (Heart) | კუნელი (K'uneli) | შავი (Shavi Khev.) |  | Fruit | Forest |
| *Crataegus pentagyna* Waldst. | 129 | Food (Human food, Tea); Medicinal (Heart, Hypertension, Tea); Utensils and tools (Dye) | კუნელი (K'uneli), შავი კუნელი (Shavi k’uneli) | კუნელი (Tsentsi Svan.), შავი (Shavi Khev.) |  | Flower, Fruit | Forest, Garden |
| *Cydonia oblonga* L. | 138 | Food (Human food); Medicinal (Bleeding, Increases haemoglobin) | კომში (K’omshi) | (Tsamala Arm.) |  | Fruit, Leaf | Garden |
| *Filipendula ulmaria* L. Mill. | 158 | Medicinal (Cold) | ქაფურა (Kapura) |  |  | Root, Whole plant | Forest |
| *Fragaria vesca* L. | 160 | Food (Human food, Pickled, Tqhemali) | მარწყვი (Marts'q'vi), ტყის მარწყვი (Tq’is marts’q’vi) | ცხეკი ხილ (Tskheki khil Svan.) |  | Fruit | Forest |
| *Fragaria vesca* L. "Alibaba" | 161 | Food (Human food) | მოცხარი (Motskhari) |  |  | Fruit | Garden |
| *Fragaria virginiana* Mill. | 162 | Food (Human food) | ხენდრო (Khendro) |  |  | Fruit | Forest, Garden |
| *Fragaria* x *ananassana* Duchesne ex Rozier | 163 | Food (Human food) | მარწყვი (Marts'q'vi) | ბასყ (Basqh Svan.) |  | Flower, Fruit | Garden |
| *Malus domestica* L. | 304 | Food (Alcohol, Human food, Svan salt, Vinegar); Medicinal (Panacea) | ვაშლი (Vashli) | ვისგვ (Viskv Svan.) | (Chershda), (garden apples), (ice apples), (Lechkhumi sinaphi), (Lushnu sanaph), (Makhara), (Mehald sanaphi), (Phear mehald), (Qhinuli), (red), (Samepho), (Saneph), (Selap), (Sinaphi), (Tchkuta viskv), (Tetne mehald), (Tzrn mehald), ანთრო (antro), ანტონოვკა (Antonovka), ბაბასჰანა (Babashana), კეხურა (Kekhura), კიტრა (Kitra), ლელმარ (Lelmar), ლენგეჭ (Lengetch), ლუზნუვიცხ (Luznuvitskh), ნეჰალდ (Nenhald), შანპანი (champagne), შაფრან (Shaphran), ცერსდა (Tzersda), ჭუმანდიფ (Tcumandiph) | Fruit | Forest, Garden |
| *Malus orientalis* Uglizk. | 305 | Food (Alcohol, Human food) | მაჟალო (Mazhalo) |  |  | Fruit | Forest, Garden |
| *Malus pumila* Mill. var. *paradisiaca* C.K. Schneid. | 306 | Food (Human food) | სამოთხის ვაშლი (Samotkhis vashli) |  |  | Fruit | Garden |
| *Mespilus germanica* L. | 319 | Food (Human food); Utensils and tools (Furniture) | ზღმარტლი (Zghmart'li), მუშმულა (Mushmula) | ზუნტი (Zunti Svan.) |  | Fruit, Stem | Forest, Garden |
| *Padus racemosa* (Lam.) Gilib. | 333 | Food (Alcohol, Human food, Tea); Medicinal (Diarrhea); Utensils and tools (Dye) | შოთხვი (Shotkhvi) |  |  | Fruit | Forest, Garden |
| *Prunus armeniaca* L. | 366 | Food (Human food) | გარგარი (Gargari) | (Kuraga Russ.) |  | Fruit | Forest, Garden |
| *Prunus avium* L. | 367 | Cultural (Musical instruments); Food (Alcohol, Human food) | (Palantsara), ბალამწარა (Balamts'ara), ბალი (Bali) | ცხეკიშ (Tskhekish Svan.), ჰებრა (Hebra Svan.) | (black), (Gogra), (red), (white), თეთრიწითელი | Fruit, Stem | Forest, Garden |
| *Prunus cerasus* L. | 368 | Food (Alcohol, Human food) | ალუბალი (Alubali) |  | (large), (small) | Fruit | Forest, Garden |
| *Prunus divaricata* Ledeb. | 369 | Food (Alcohol, Human food, Tqhemali) | ტყემალი (Tq’emali) | ბარყვენდ (Barqhvend Svan.) | (red), (white), (yellow) | Fruit | Forest, Garden |
| *Prunus insititita* L. | 371 | Food (Human food) | ღოღნოშო (Ghoghnasho) |  |  | Fruit | Garden |
| *Prunus laurocerasus* L. | 372 | Food (Human food) | წყავი (Tz'qh'avi) |  |  | Leaf | Garden |
| *Prunus padus* L. | 373 | Utensils and tools (Tool handles) |  | მჯნელა (Mjnela Svan.) |  | Stem | Forest |
| *Prunus persica* (L.) Batsch | 374 | Food (Human food) | ატამი (Atami) |  |  | Fruit | Garden |
| *Prunus spinosa* L. | 375 | Food (Chave, Human food) | კვინჩხაი (Kvinchkhai) |  |  | Fruit | Forest, Garden |
| *Prunus vachuschtii* Bregaze | 376 | Food (Human food) | ალუჩა (Alucha) |  |  | Fruit | Garden |
| *Prunus* x *domestica* L. | 377 | Food (Alcohol, Human food) | ქლიავი (Kliavi) |  | (Alibukhari), (black), (Makhara), (Tzqhalqliava), (white), ჭანჭური (Ch'anch'uri), ჭანჭური (Shavi), ჭანჭური (Tetri) | Fruit | Garden |
| *Pyrus caucasica* Fed. | 383 | Construction (Timber); Food (Alcohol, Human food, Phkhali, Syrup); Utensils and tools (Household utensils) | პანტა (P'ant’a) |  |  | Fruit, Stem | Forest, Garden |
| *Pyrus communis* L. | 384 | Food (Alcohol, Human food) | მსხალი (Mskhali) | იცხი (Itskhi Svan.) | (Aqhari), (Gulabi), (Guril), (Gvirkesh), (Katsitava), (Kefri), (Khetchetchuri), (Lelqhin), (Luznu), (Makhali), (Malintu), (Phkhantchaash), (Shavmskhala), (Tchkuta vitskh), (Tchumend), (Tsument), ბაბანი (Baban), ბაბაროზან (Babarozan), ბაბენ (Baben), გობრუ (Gobru), გომბრო (Gombro), ვირკეშ (Virkesh), თეთნო (Tetno), კიფერ (Kipher), ლელმარ (Lelmar), ლეფხანჭ (Lephkhantch), ლოვყენ (Lovqhen), ლოზმ (Lozm), მანინტ (Manint), მაშკვანი (Maskhvan), მირკეშ (Mirkesh), ნენსგაშვიხ (Nensgashvikh), ხანჭაშ ცალან (Khantchash tsalan) | Fruit | Forest, Garden |
| *Rosa canina* L. | 403 | Food (Alcohol, Beer, Human food, Marmalade, Tea); Medicinal (Vitamins) | ასკილი (Ask'ili) |  |  | Fruit | Forest |
| *Rosa pimpinellifolia* Boiss. | 404 | Food (Beer, Human food, Tea) | ასკილი (Ask'ili), შავი ასკილი (Shavi Ask’ili) |  |  | Fruit | Forest |
| *Rosa* sp. | 405 | Food (Alcohol, Human food, Tea); Medicinal (Cold, Cough, Gallbladder, Kidneys, Tea) | ასკილი (Ask'ili), ვარდი (Vardi) | ხვარ (Khvar Svan.) |  | Flower, Fruit, Leaf | Forest, Garden |
| *Rubus fruticosus* L. | 407 | Food (Human food) | მაყვალი (Ma'qvali), რუსული მაყვალი (Rusuli maq’vali) | Малина (Malina Russ.) |  | Fruit | Forest, Garden |
| *Rubus idaeus* L. | 408 | Food (Human food, Tea); Medicinal (Cold, Wounds) | ჟოლო (Zholo) | (Khvapa Tush.), Малина (Malina Russ.), ინღა (Ingha Svan.), ხვაფა (Khvapa Tush.) |  | Flower, Fruit, Leaf | Forest, Garden |
| *Rubus saxatilis* L. | 409 | Food (Chave, Human food) | ჟოლის-დედა (Zholis-deda), ხახამა (Khakhama) | მწყერთიფქლა (Mts'q'ertipkla Khev.), წერტიფხლა (T'sert'ipkhla Khev.) |  | Fruit | Forest |
| *Rubus* sp. | 410 | Food (Human food, Wine); Medicinal (Anemia, Cold, Inflammation) | მაყვალი (Maqhvali) | ვიღვი (Vighv Svan.), უღვ (Ughv Svan.) |  | Fruit, Leaf | Forest, Garden |
| *Sorbus boissieri* C.K. Schneid. | 444 | Food (Human food); Medicinal (Hypertension) | ცირცელი (Tsirtseli) |  |  | Fruit | Forest |
| *Sorbus caucasigena* Kom. | 445 | Food (Alcohol, Beer, Human food, Marmalade); Medicinal (Blood pressure, Cramps, Heart, Hypertension, Wounds); Utensils and tools (Household utensils, Tool handles) | ცირცელი (Tsirtseli) | გოგლანდ (Gogland Svan.) |  | Fruit, Leaf, Stem | Forest, Garden |
| *Sorbus terminalis* Crantz. | 446 | Food (Human food); Utensils and tools (Furniture) | თამელი (Tameli) | მურგვი (Murgvi Svan.) |  | Fruit, Stem | Forest |
| *Sorbus torminalis* C. Crantz. | 447 | Food (Human food) | დათვისყურა (Datvisqhura) |  |  | Fruit | Forest |
| *Spiraea hypericifolia* L. | 450 | Utensils and tools (Brooms) | გრაკლა (Grak'la) | მაკაცი (Makatzi Khev.) |  | Stem | Forest |
| **Rubiaceae** |  |  |  |  |  |  |  |
| *Rubia tinctorum* L. | 406 | Utensils and tools (Dye) | ენდრო (Endro) |  |  | Fruit, Root, Whole plant | Forest |
| **Russulaceae** |  |  |  |  |  |  |  |
| *Lactarius deliciosus (*L. ex Fr.) S.F. Grey | 277 | Food (Human food) | მჭადა (Mtchada) | ჭადუა (Tchadua Svan.) |  | Fruit | Forest |
| *Lactifluus piperatus* (L.) Roussel | 278 | Food (Human food, Pickled) |  | ბერუითავი (Beruitavi Svan.) |  | Fruit | Forest |
| *Lactifluus volemus (*Fr.) Kuntze | 279 | Food (Human food) | მჭადა (Mch'ada) |  |  | Fruit | Forest |
| *Russula emetica (*Schaeff.) Pers. | 418 | Food (Human food) | ბღავანა (Bahgavana) |  |  | Fruit | Forest |
| *Russula rosea* Pers. | 419 | Food (Human food) | წითლიო (Ts'itlio) |  |  | Fruit | Forest |
| *Russula virescens* (Schaeff.) Fr. | 420 | Food (Human food) | ხახვილო (Khalhvilo) |  |  | Fruit | Forest |
| **Rutaceae** |  |  |  |  |  |  |  |
| *Citrus limon* (L.) Burm. f. | 111 | Food (Human food) | ლიმონი (Limoni) |  |  | Fruit | Garden |
| *Citrus* x *paradisi* Macfad. | 112 | Food (Human food) | გრეიპფრუტი (Greip'prut'i) |  |  | Fruit | Garden |
| *Citrus* x *sinensis* L. | 113 | Food (Human food) | ფორთოხალი (Portokhali) |  |  | Fruit | Garden |
| **Salicaceae** |  |  |  |  |  |  |  |
| *Populus tremula* L. | 361 | Construction (Timber); Cultural (Musical instruments); Medicinal; Utensils and tools (Household utensils, Tool handles, Vessels) | ვერხვი (Verkhvi) |  |  | Leaf, Stem | Forest |
| *Salix alba* L. | 421 | Construction (Timber); Utensils and tools (Baskets, Tool handles) | ტირიფი (Tiriphi) |  |  | Stem | Forest |
| *Salix caprea* L. | 422 | Animal food (Fodder); Construction (Fences, Timber, Walls); Medicinal (Arthritis, Gallstones, Kidneys); Utensils and tools (Baskets, Bows, Snowshovels, Tool handles, Tough utensils) | მდგნალი (Mdgnali) | ბაგუნდი (Bagund Svan.), ფოხვი (Pokhvi tush.), ფოხვი (Pokhvi tush.), ჭიჭუნი (Chitchuni Svan.) |  | Bark, Branches, Leaf, Stem | Forest |
| **Sapindaceae** |  |  |  |  |  |  |  |
| *Acer campestre* L. | 3 | Construction (Timber); Utensils and tools (Furniture, Tool handles) | ნეკერჩხალი (Nek'erchkhali) |  |  | Stem | Forest |
| *Acer platanoides* L. | 4 | Utensils and tools (Furniture, Household utensils, Sleds, Tool handles) | ნეკერჩხალი (Nek'erchkhali) | თეკრა (Tekra Svan.) |  | Stem | Forest |
| Acer pseudoplatanus L. | 5 | Food (Tea); Utensils and tools (Plane, Spinning wheels) | მთის ბოყვი (Mtis bok’vi), ნეკერჩხალი (Nek'erchkhali) | თეკრა (Tekra Svan.) |  | Flower, Stem | Forest |
| *Acer trautvetteri* Medw. | 6 | Construction (Timber); Utensils and tools (Barrels, Bowls, Household utensils, Spoons, Tool handles, Trays, Vessels) | ბოკვი (Bok'vi), თეკრი (Tekri) |  |  | Stem | Forest |
| **Scrophulariaceae** |  |  |  |  |  |  |  |
| *Verbascum* sp. | 487 | Medicinal (Gallbladder, Haemorrhoids, Kidneys, Skin problems) | ქერიფქლა (Keripkla) |  |  | Flower, Leaf | Forest |
| **Smilacaceae** |  |  |  |  |  |  |  |
| *Smilax excelsa* L. | 439 | Food (Human food, Phkhali) | ეკალღიჭი (Ek’alghich’i) | ეკალა (Ek'ala) |  | Branches, Leaf, Stem | Forest |
| **Solanaceae** |  |  |  |  |  |  |  |
| *Capsicum annuum* L. | 89 | Food (Human food, Pickled, Svan salt); Medicinal (Cold, Flu) | წიწაკა (Ts'tsak'a) | მწარე წიწაკა (Mts'are ts'its'ak'a Khev.) | (chili), (sweet) | Fruit | Forest, Garden |
| *Capsicum annuum* L. sweet Bulgarian | 90 | Food (Human food, Svan salt) | წიწაკა წითელი (Tzitzaka tziteli) | ძაფანა (dzaphana Svan.) |  | Fruit | Garden |
| *Hyoscyamus niger* L. | 217 | Cultural (Hallucinogenic); Medicinal (Anti-fungal, Toothache) | ლენცოფა (Lentsopa), საპინა (Sap’ina) |  |  | Leaf, Seed | Forest |
| *Lycopersicum esculentum* L. | 301 | Food (Human food, Pickled) | პამიდორი (P'amidori) |  |  | Fruit, Stem | Garden |
| *Nicotiana rustica* L. | 325 | Cultural (Masticant, Smoking, Snuff); Medicinal (Arthritis, Bronchitis, Sore throat, Tonsilitis, Wounds) | ბურნუთი (Burnuti), წეკო (Ts'ek'o) | თუთინ (Tutin Svan.), წეკვა (Tsekva Khev.) |  | Leaf, Stem | Forest, Garden |
| *Nicotiana tabacum* L. | 326 | Cultural (Smoking); Medicinal (Arthritis, Bronchitis, Digestive system, Sinusitis, Skin problems); Utensils and tools (Natural pesticide) | ბურნუთი (Burnuti), წეკო (Ts'ek'o), ჯოკარი (jokari) | თუთინ (Tutin Svan.), წეკვა (Tsekva Khev.) |  | Leaf, Whole plant | Forest, Garden |
| *Physalis alkekengi* L. | 343 | Food (Human food) | ონტკოფა (Ont’k’opa) |  |  | Leaf | Forest |
| *Solanum melogena* L. | 440 | Food (Human food) | ბადრიჯანი (Badrijani) |  |  | Fruit, Leaf | Garden |
| *Solanum nigrum* L. | 441 | Medicinal (Toothache) | ძაღლყურძენა (Dzaghlq’urdzena) |  |  | Stem | Forest |
| *Solanum tuberosum* L. | 442 | Food (Alcohol, Chachapuri, Human food, Phkhali, Pickled) | კარტოფილი (K'art'opili) |  | (red), (white) | Fruit, Leaf, Root, Stem | Garden |
| **Sparassidaceae** |  |  |  |  |  |  |  |
| *Sparassis crispa* Wulfen | 448 | Food (Human food) | კომბუოსტოსოკო (Kombostosoko) |  |  | Fruit | Forest |
| **Staphyleaceae** |  |  |  |  |  |  |  |
| *Staphylea colchica* Steven | 451 | Food (Human food, Phkhali) | ჯონჯოლი (Jonjoli) | ნიორკავა (Niorkava Svan.) |  | Flower, Fruit, Root | Forest, Garden |
| **Suillaceae** |  |  |  |  |  |  |  |
| *Suillus granulatus* (L.) Roussel | 452 | Food (Human food) | დუმა სოკო (Duma soko) | Маслята (Masliata Russ.) |  | Fruit | Forest |
| *Suillus luteus* (L.) Roussel | 453 | Food (Human food) | ზეთიანა (Zetiana) | Маслята (Masliata Russ.) |  | Fruit | Forest |
| **Taxaceae** |  |  |  |  |  |  |  |
| *Taxus baccata* L. | 460 | Food (Human food); Utensils and tools (Tool handles) | უთხოვარი (Utkhovari), ურთხელი (Urtkheli) |  |  | Fruit, Stem | Forest |
| **Theaceae** |  |  |  |  |  |  |  |
| *Camelia sinensis* L. | 82 | Food (Tea) | ჩაი (Chai) |  |  | Fruit | Garden |
| **Thymeleaceae** |  |  |  |  |  |  |  |
| *Daphne caucasica* Pall. | 140 | Medicinal (Toothache); Veterinary (Ektoparasites) | ზარავანდი (Zaravandi) | ზარავანდი (Majaghveri Khev.) |  | Stem | Forest |
| *Daphne glomerata* L. | 141 | Medicinal (Toothache) | წიბა (Ts'iba) | მოგოცხარა? (Magozhara Khev.) |  | Leaf | Forest |
| *Daphne mezereum* L. | 142 | Medicinal (Toothache) | მაჯაღვერი (Majaghveri), წიბლა (Ts’ibla) | მოგოცხარა? (Magozhara Khev.) |  | Leaf | Forest |
| *Daphne pontica* L. | 143 | Medicinal (Tea) |  | ზარავანდი (Majaghveri Khev.) |  | Leaf | Forest |
| **Tricholomataceae** |  |  |  |  |  |  |  |
| *Lepista sordida* (Schumach.) Singer | 291 | Food (Human food) | ღრუბელა, მელნისძირა, მელანო - (Ghrubela, Melnisdzira, Melano) |  |  | Fruit | Forest |
| *Tricholoma aurantium* (Schaeff.) Ricken | 469 | Food (Human food) | ხოხბის მკერდი (Khokhbis mk'erdi) |  |  | Fruit | Forest |
| *Tricholoma portentosum (*Fr.) Quél. | 470 | Food (Human food) | შავჩოხა (Shavchokha) |  |  | Fruit | Forest |
| **Ulmaceae** |  |  |  |  |  |  |  |
| *Celtis caucasica* Willd. | 96 | Utensils and tools (Spindles) | აკაკის ხე (Akakis-khe) |  |  | Stem | Forest |
| *Ulmus elliptica* C. Koch | 477 | Construction (Timber); Utensils and tools (Carts, Furniture) | თელა (Tela) |  |  | Stem | Forest |
| *Ulmus glabra* Huds. | 478 | Construction (Timber) | თელა (Tela) |  |  | Branches | Forest |
| **Urticaceae** |  |  |  |  |  |  |  |
| *Urtica dioica* L. | 479 | Animal food (Fodder); Food (Beverage, Chachapuri, Chave, Human food, Khinkali, Phkhali, Pickled); Medicinal (Antiseptic, Burns, Hypertension, Increases haemoglobin, Joints, Nerves, Wounds) | ჭინჭარი (Ch’inch’ari) | (Santachi Arm.), მერხელ (Merkhel Svan.) |  | Leaf, Stem | Forest, Garden |
| **Valerianaceae** |  |  |  |  |  |  |  |
| *Valeriana officinalis* L. | 485 | Food (Tea); Medicinal (Cold, Cough, Heart, Nerves, Pleuritis, Pneumonia) | კატაბალახა (Katabalakha), კატაბალახა (K'at'abalakha) | გულბანდი (Gulbandi Tush.) |  | Leaf, Root | Forest |
| **Violaceae** |  |  |  |  |  |  |  |
| *Viola arvensis* L. | 492 | Food (Phkhali) | პატარძალა (P'at'ardzala) |  |  | Leaf | Forest |
| *Viola* sp. | 493 | Food (Chachapuri, Pickled) | ია (Ia) | ია-ია (Ia-ia) |  | Leaf, Root | Forest |
| **Viscaceae** |  |  |  |  |  |  |  |
| *Viscum album* L. | 494 | Medicinal (Heart, Hypertension) | ფითრი (Pitri) |  |  | Leaf | Forest |
| **Vitaceae** |  |  |  |  |  |  |  |
| *Vitis vinifera* L. | 495 | Food (Alcohol, Human food); Medicinal (Anemia, Blood pressure increase, Seizures prevent) | ვაზი (Vazi), ყურძენი (Qhurdzeni), ყურძენი (Q'udzeni), ხეჭეჭური (Ch'etsheturi) |  |  |  |  |
